# A self-oxygenating polyphenol-nanozyme hydrogel remodels the inflammatory microenvironment for diabetic wound healing

**DOI:** 10.1093/rb/rbag118

**Published:** 2026-06-12

**Authors:** Shangqing Huang, Yujia Zheng, Shengxi Jiang, Huabin Liu, Lijun Tang, Chaoming Xie, Jie Weng, Yanan Jiang

**Affiliations:** Key Laboratory of Advanced Technologies of Materials Ministry of Education, Institute of Biomedical Engineering, College of Medicine, Southwest Jiaotong University, Chengdu 610031, China; School of Materials Science and Engineering, Southwest Jiaotong University, Chengdu 610031, China; Department of Gastroenterology, Chengdu Integrated TCM & Western Medicine Hospital, Chengdu 610059, China; Key Laboratory of Advanced Technologies of Materials Ministry of Education, Institute of Biomedical Engineering, College of Medicine, Southwest Jiaotong University, Chengdu 610031, China; School of Materials Science and Engineering, Southwest Jiaotong University, Chengdu 610031, China; Key Laboratory of Advanced Technologies of Materials Ministry of Education, Institute of Biomedical Engineering, College of Medicine, Southwest Jiaotong University, Chengdu 610031, China; School of Materials Science and Engineering, Southwest Jiaotong University, Chengdu 610031, China; Key Laboratory of Advanced Technologies of Materials Ministry of Education, Institute of Biomedical Engineering, College of Medicine, Southwest Jiaotong University, Chengdu 610031, China; School of Materials Science and Engineering, Southwest Jiaotong University, Chengdu 610031, China; Key Laboratory of Advanced Technologies of Materials Ministry of Education, Institute of Biomedical Engineering, College of Medicine, Southwest Jiaotong University, Chengdu 610031, China; School of Materials Science and Engineering, Southwest Jiaotong University, Chengdu 610031, China; Department of General Surgery & Tissue Stress Injury and Functional Repair Key Laboratory of Sichuan Province, The General Hospital of Western Theater Command (Chengdu Military General Hospital), Chengdu 610083, China; Key Laboratory of Advanced Technologies of Materials Ministry of Education, Institute of Biomedical Engineering, College of Medicine, Southwest Jiaotong University, Chengdu 610031, China; School of Materials Science and Engineering, Southwest Jiaotong University, Chengdu 610031, China; Key Laboratory of Advanced Technologies of Materials Ministry of Education, Institute of Biomedical Engineering, College of Medicine, Southwest Jiaotong University, Chengdu 610031, China; School of Materials Science and Engineering, Southwest Jiaotong University, Chengdu 610031, China; Obesity and Metabolism Medicine-Engineering Integration Laboratory, Department of General Surgery, The Affiliated Hospital of Southwest Jiaotong University, The Third People’s Hospital of Chengdu, Chengdu 610031, China; Medical Research Center, The Third People’s Hospital of Chengdu, Affiliated Hospital of Southwest Jiaotong University, Chengdu 610014, China

**Keywords:** immunomodulatory hydrogels, self-oxygenating, polyphenol-nanozyme, diabetic wound healing

## Abstract

Diabetic wounds are characterized by prolonged hyperglycemia and associated hypoxia, induce oxidative stress and sustained inflammation, which collectively impair angiogenesis and hinder the healing process. Conventional oxygen‑supplying hydrogel dressings are often limited by inefficient oxygen release, poor stability, and insufficient multifunctionality. To overcome these limitations, we have designed a GelMA–MnPZC multifunctional nanocomposite hydrogel dressing capable of stable and prolonged oxygen release. This hydrogel remodels the hypoxic and inflammatory wound microenvironment through sustained oxygen generation and polyphenol‑mediated activity, thereby creating favorable conditions for angiogenesis and tissue regeneration. The hydrogel was fabricated by embedding polydopamine–manganese (PDA–Mn) nanozyme‑modified, ZIF‑8‑encapsulated CaO_2_ nanoparticles into a gelatin-methacryloyl (GelMA) network. The ZIF‑8 shell enhances the stability of CaO_2_, while the PDA‑Mn nanozyme exhibits catalase‑like activity, together ensuring a stable and efficient self‑oxygenating capability under hypoxic conditions. Furthermore, the hydrogel’s excellent tissue adhesion ensures long-term stable wound management. Additionally, sustained release of Zn^2+^ and Mn^2+^ ions provide potent antibacterial activity, reducing the risk of wound infection. In summary, the GelMA–MnPZC hydrogel accelerates diabetic wound healing through a coordinated multifunctional mechanism, including antibacterial action, oxidative stress mitigation, hypoxia alleviation, and immunomodulation. This innovative self‑oxygenating hydrogel represents a promising clinical strategy for the comprehensive treatment of diabetic wounds.

## Graphical Abstract

**Graphical Abstract:**
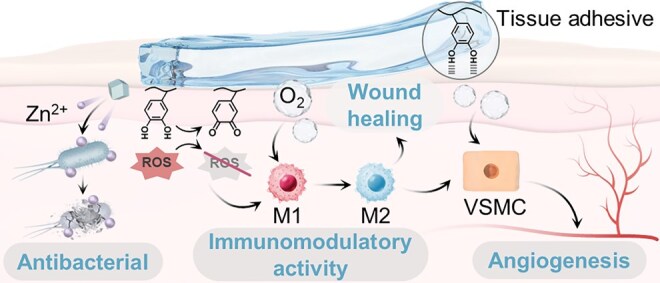

## Introduction

Diabetic skin wounds pose a major clinical challenge because their healing is impaired by a pathological microenvironment characterized by excessive oxidative stress, persistent inflammation, vascular dysfunction and tissue hypoxia [1, 2]. The hypoxic milieu plays a particularly detrimental role in tissue repair. Oxygen is essential for multiple physiological processes critical to healing, including immune defense, cellular proliferation, angiogenesis, collagen deposition and tissue remodeling [3, 4]. First, immune cells rely on oxygen to generate reactive oxygen species (ROS) for effective bacterial clearance; hypoxia severely undermines this oxygen-dependent antimicrobial activity, thereby increasing susceptibility to wound infection [5]. Second, as the primary substrate for cellular energy production, oxygen deficiency leads to an intracellular ‘energy crisis’, resulting in functional impairment [6]. Moreover, sustained metabolic dysfunction exacerbates oxidative stress, which in turn perpetuates a state of chronic inflammation [7]. This persistent inflammatory response further damages endothelial function and inhibits angiogenesis, collectively obstructing the healing cascade [8]. Consequently, the development of wound dressings that can actively ameliorate the hypoxic and inflammatory microenvironment holds considerable promise for advancing diabetic wound therapy.

Hydrogels are considered an ideal choice as dressing materials for skin wounds due to their excellent flexibility, skin compatibility modulus and superior moisturizing properties [9, 10]. However, conventional hydrogel dressings often lack multifunctionality and are unable to generate oxygen to alleviate the hypoxic microenvironment of chronic wounds. To address this issue, various oxygen-generating materials (such as hemoglobin, peroxides and algae) have been integrated into hydrogels [3, 11, 12]. Among these, calcium peroxide (CaO_2_), a relatively stable member of the peroxide family, has garnered significant attention owing to its easy availability, straightforward storage and transportation, and rapid oxygen-release capability [13]. Nevertheless, the oxygen production of CaO_2_ still depends on its spontaneous decomposition in water, which generates hydrogen peroxide (H_2_O_2_) that subsequently decomposes into oxygen [14, 15]. This reaction tends to be rapid and uncontrolled, resulting in a burst release that fails to provide sustained and stable oxygen delivery [14, 16]. Moreover, the spontaneous decomposition of H_2_O_2_ exhibits low oxygen-production efficiency and uncontrollably yields hydroxyl radicals (•OH), which can aggravate oxidative stress in the wound tissue [3]. Therefore, functionalizing CaO_2_ to develop hydrogel dressings capable of continuous and stable oxygen release is of critical importance. Recently, metal–organic frameworks (MOFs) have shown great promise as effective vehicles for the sustained and controlled release of active agents [17, 18]. Among various MOFs, zeolitic imidazolate framework-8 (ZIF-8) stands out as an excellent candidate for stabilization, thanks to its large specific surface area, high loading efficiency and pH-responsive behavior [19–21]. In this work, we propose to achieve stable and prolonged oxygen release under mild physiological conditions by *in situ* immobilization of CaO_2_ within a ZIF-8 matrix, thereby constructing a ZIF-8-armored CaO_2_ complex for next-generation hydrogel-based wound dressings.

Inflammation serves as a vital component of the physiological healing cascade, primarily responsible for clearing cellular debris and potential pathogens from the wound site [22, 23]. However, in diabetic wounds, the chronic hyperglycemic and pro-oxidative microenvironment excessively amplifies the inflammatory response [24]. As a result of this dysregulation, macrophages are unable to effectively transition from their pro-inflammatory (M1) state to the anti-inflammatory (M2) state [25]. Accordingly, elevated levels of inflammatory mediators such as IL-6, TNF-α and iNOS persist, which in turn disrupts the orderly transition of diabetic wounds from the inflammatory to the proliferative phase, leading to a marked delay in overall wound healing [2, 26]. Thus, the capacity of a wound dressing to actively modulate the inflammatory microenvironment is crucial for effective diabetic wound repair.

Polyphenolic materials have demonstrated outstanding antioxidant and immunomodulatory properties [27, 28]. In addition, emerging studies have revealed that the interfacial coupling of phenolic compounds with quinone-based nucleophilic groups on diverse substrates offers a promising strategy for constructing robust bioadhesives [29, 30]. Additionally, owing to their abundant catechol functional groups, polyphenolic structures can be flexibly coordinated with metal ions to form polyphenol–metal nanozymes with enzyme-like activities [31]. The robust catalase-like activity inherent to these nanozymes presents an attractive strategy to augment the oxygen evolution efficiency of CaO_2_-based platforms.

**Figure 1:**
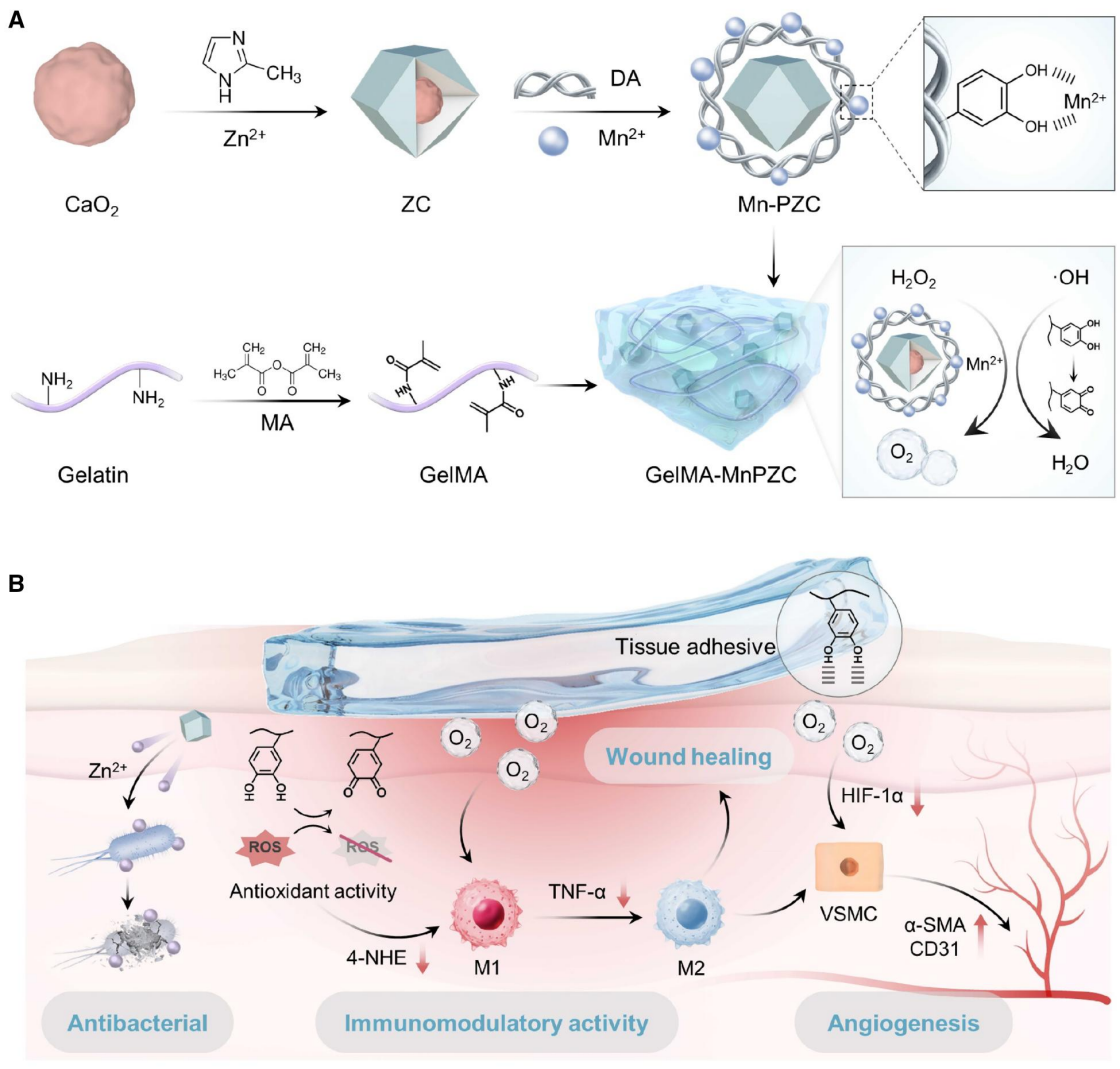
Schematic illustration of the preparation and working mechanism of the GelMA–MnPZC hydrogel for promoting hypoxic diabetic wound healing. (**A**) Synthesis of the GelMA–MnPZC hydrogel. (**B**) GelMA–MnPZC hydrogel provides highly effective antibacterial action and synergistically remodels the hypoxic and inflammatory microenvironment, accelerating the healing of diabetic wounds.

In this work, we engineered a multifunctional nanocomposite hydrogel dressing with sustained oxygen-release capacity, designed through polyphenol-based chemistry to actively modulate the hypoxic and inflammatory microenvironment of diabetic wounds and accelerate healing (Figure 1). Our strategy involved three principal steps. First, a ZIF-8-armored CaO_2_ composite nanoparticle (ZC) was synthesized via the *in situ* encapsulation of CaO_2_ within a ZIF-8 matrix. Subsequently, a polydopamine–manganese (PDA–Mn) nanozyme was integrated as a catalytic mediator, yielding a PDA-Mn/ZIF-8@CaO_2_ nanoreactive system (MnPZC). Finally, the MnPZC nanoparticles were uniformly incorporated into a methacrylated gelatin (GelMA) hydrogel network, which mimics the native extracellular matrix, thus forming the GelMA–MnPZC nanocomposite hydrogel. The encapsulation of CaO_2_ within ZIF-8 significantly bolstered its stability, endowing the hydrogel with the capacity for controlled and sustained oxygen delivery. Meanwhile, the incorporated PDA-Mn nanozyme acted as a catalase mimic, effectively boosting the oxygen-generation efficiency of the system. In addition, the catechol-rich PDA component conferred robust tissue-adhesive properties to the hydrogel. Further, the gradual degradation of the composite allowed sustained release of bioactive Zn^2+^ and Mn^2+^ ions, imparting potent antibacterial activity. Collectively, the GelMA–MnPZC hydrogel promotes wound healing through multiple coordinated mechanisms: efficient antibacterial action, stable oxygen supply and mitigation of oxidative stress and inflammation. By modulating the deleterious hypoxic and inflammatory milieu characteristic of diabetic wounds, this multifunctional dressing represents a novel and promising advanced platform for therapeutic intervention in diabetic wound healing.

## Materials and methods

### Materials

All chemical reagents of analytical grade were commercially sourced and used without further purification. Specifically, gelatin, methacrylic anhydride (MA) and dopamine were obtained from Sigma-Aldrich (USA); manganese chloride, sodium hydroxide, zinc nitrate, methanol, anhydrous calcium chloride, hydrogen peroxide, anhydrous ethanol and ammonia from Kelong (Chengdu, China); and 2-methylimidazole (HmIM), MA, ammonium persulfate (APS), tetramethylethylenediamine (TMEDA), along with other reagents, from Mecolin (China).

### Preparation of MnPZC

1. Preparation of CaO_2_: The CaO_2_ and ZIF-8 MOFs were fabricated according to the previously reported method. About 1 g CaCl_2_ was dissolved in 5 mL of deionized water, then it was added to a beaker containing 60 mL of anhydrous ethanol and was stirred vigorously for 10 min. To the above solution 5 mL of H_2_O_2_ solution (30% by mass) was added under stirring for 2 min, followed by the slow addition of 5 mL of 1 mol/L ammonia solution and further stirring for 30 min. After washing the mixture three times with anhydrous ethanol, the precipitates were collected and freeze-dried to yield CaO_2_ powder.
2. Preparation of ZC: About 0.45 g of Zn (NO_3_)_2_ and 1 g of dimethylimidazole were separately dissolved in 2 mL of methanol. Then 0.1 g of CaO_2_ nanoparticles was dispersed in the dimethylimidazole methanol solution, stirred at 500 rpm and sonicated at 100 W, 40 kHz for 10 min. Finally, the resulting precipitates were collected by centrifugation (10 000 rpm, 10 min), washed three times with methanol and freeze-dried to obtain the ZC nanoparticles.
3. Preparation of MnPZC: After dissolving 5 mg of dopamine in 5 mL of water, 5 mg of MnCl_2_ was added with stirring until fully dissolved. Subsequently, 100 μL of NaOH solution (0.5 g/mL) was introduced and stirred for 5 min. Meanwhile, 0.2 g of ZC nanoparticles was dispersed in 15 mL of ethanol. The resulting dispersion was then combined with the previous solution and stirred for 10 min. The final precipitates were isolated by centrifugation to yield MnPZC nanoparticles.

### Characterization of MnPZC

1. Microstructure and crystal structure characterization of nanoparticles: The morphology of the CaO_2_, ZIF-8, ZC and MnPZC was observed by a scanning electron microscope (SEM, JSM 6390, JEOL, Japan). The morphology and structure of MnPZC nanoparticles were imaged using transmission electron microscopy (TEM, JSM 2100F, JEOL, Japan). The elemental distribution of the MnPZC nanoparticles was measured using energy-dispersive X-ray spectroscopy (EDS). The crystal phases of the CaO_2_, ZIF-8, ZC and MnPZC were determined by X-ray diffraction (XRD, X’pert PRO, Philips, The Netherlands) over the 2*θ* range of 5°–60° at a rate of 0.1°/s. The chemical states of the nanoparticles were assessed by X-ray photoelectron spectroscopy (ESCALAB 250Xi, Thermo Scientific, USA).
2. Zeta potential analysis of nanoparticles: Ethanol solutions of ZC and MnPZC nanoparticles were analysed potentiometrically using a multifunctional electroacoustic Zeta potential analyser. The electrophoresis cups were rinsed with anhydrous ethanol between measurements to prevent cross-contamination.
3. Brunauer–Emmett–Teller (BET): N_2_ adsorption/desorption isotherms were measured at 77 K on an ASAP 2460 system. Samples were vacuum-outgassed at 120°C for 24 h before measurement. Ultrahigh purity gases (99.999%) were used throughout. Apparent surface areas were calculated from the nitrogen adsorption data via the BET method.
4. Atomic absorption spectroscopy: A certain amount of MnPZC powder was dispersed in RO water, and glacial acetic acid was added to adjust its pH to acidic so that all MnPZC was dissolved. The content ratio of Zn, Mn and Ca elements in the solution was determined by atomic absorption spectrometry (Z-2300, Hitachi, Japan), thereby calculating the content of each element in MnPZC.
5. Catalase activity: About 50 mg MnPZC and ZC were, respectively, incubated with 25 mL 10 mM H_2_O_2_ in PBS at 37°C, and dissolved oxygen was monitored in real time using a portable dissolved oxygen meter. About 10 mM H_2_O_2_ in PBS without nanoparticles was used as a blank control. Tests were performed for 5, 10, 15 and 20 min.
6. Oxygen release curve: Each test sample (0.1 g of CaO_2_, ZC or MnPZC nanoparticles) was added to 100 mL of deionized water pre-purged with nitrogen to remove dissolved oxygen. Oxygen generation was measured at 37°C using a dissolved oxygen meter (JB608, LeiMagnet, Shanghai) at regular intervals. Each measurement was recorded for 1 min until a stable reading was obtained. The dissolved oxygen content in the water in real time was used as a control. Oxygen production = oxygen content of the sample − oxygen content of the control.

### Preparation of gelatin methacrylate

The gelatin methacrylate (GelMA) was prepared according to our previously reported study [19]. Briefly, 20 g fish gelatin were first dissolved in 200 mL of PBS (pH = 7.4) at 60°C. To the gelatin solution was slowly added 16 mL of MA, and the mixture was stirred at 50°C for 3 h. The reaction was stopped by adding 200 mL of PBS. The solution was then dialyzed (MWCO 14 000) against deionized water at 40°C for 7 days, with water changes every 24 h. The dialysate was freeze-dried to obtain GelMA.

### Preparation of hydrogels

1. GelMA hydrogel: The GelMA hydrogel was prepared *via* the following steps. GelMA (2 g) was dissolved in 7 mL of deionized water at 60°C. The hydrogel precursor solution was prepared by adding 200 μL PEGDA, 0.3 g of APS and 20 μL of TMEDA into the GelMA solution. The mixture was allowed to gel at room temperature, forming GelMA hydrogels.
2. GelMA–ZC hydrogel: GelMA (2 g) was dissolved in 7 mL of DI water at 60°C. About 0.1 g ZC was dispersed into 2 mL DI water and sonicated for 10 min. Then, ZC solution was added to GelMA solution with continuous stirring. Finally, the hydrogel precursor solution was prepared by adding 200 μL of PEGDA, 0.3 g of APS and 20 μL of TMEDA into the GelMA solution. The mixture was allowed to gel at room temperature, forming GelMA–ZC hydrogels.
3. GelMA-MnPZC hydrogel: The GelMA-MnPZC hydrogel was prepared via the following steps. About 2 g of GelMA was dissolved in 7 mL deionized water at 60°C. About 0.1 g MnPZC was dispersed into 2 mL DI water and sonicated for 10 min. Then, MnPZC solution was added to GelMA solution with continuous stirring. Finally, the hydrogel precursor solution was prepared by adding 200 μL of PEGDA, 0.3 g of APS and 20 μL of TMEDA into the GelMA solution. The mixture was allowed to gel at room temperature, forming GelMA-MnPZC hydrogels.

To investigate the effect of different MnPZC nanoparticles on the adhesion properties of hydrogels, this study synthesized hydrogels with different MnPZC nanoparticle contents (0.05 wt.%, 0.1 wt.%, 0.2 wt.%) using a similar method.

### Microstructural characterizations of the hydrogels

The microstructure and elemental mapping of the hydrogels (GelMA, GelMA–ZC and GelMA–MnPZC) were examined with the SEM (JSM 6390, Japan) equipped with an energy dispersive spectrometer. The hydrogels were freeze-dried, and then cut with a razor blade to expose the cross-sectional surface prior to observation. The pore structure of the hydrogels was visualized by SEM after gold sputter coating.

### Adhesion properties of hydrogels

Hydrogel specimens (10 mm × 10 mm × 2 mm) were sandwiched between two pigskin substrates. Adhesion was measured using a universal testing machine at 2 mm/min until detachment occurred. Adhesion strength (As) was calculated as As = *F*/*A*, with *F* being the maximum load and *A* the sample area.

### Mechanical properties of hydrogels

#### Tensile testing

Tensile tests were performed using a universal testing machine (UTM, Instron 5567, USA) at a loading rate of 2 mm/min. A 2 mm thick hydrogel was cut into strips (25 mm long, 25 mm wide). The caliper length between the clamps was 5 mm. Samples were mounted with a 5 mm gauge length and pulled at 2 mm/min (*n* = 5 per group) until mid-sample fracture. Cyclic tensile tests were conducted for 10 cycles at a maximum strain of 50% of the original length. The tensile force (*F*) was divided by the cross-sectional area (*A*) to obtain the tensile stress (*σ*), *σ* = *F*/*A*. The tensile strain (*ε*) was obtained by dividing the tensile length (Δ*L*) by the original length (*L*_0_), calculated using the formula:


$$\varepsilon (\%)=\frac{L-L_{0}}{L_{0}}\;\times 100\%$$

The elongation (*λ*) is obtained by dividing the deformed length (*L*) by the original length (*L*_0_), and the calculation is shown in the formula:


$$\lambda (\%)=\frac{L}{L_{0}}\times 100\%$$

#### Compression test

Hydrogel specimens (15 mm × 15 mm × 11 mm) were compressed at 5 mm/min to 50% strain (*n* = 5 per group). Cyclic compression was conducted over 10 cycles (computer-controlled) at a maximum strain of 50% of original height. Compressive strength was calculated as *P* = *F*/*S*, where *F* = compressive load and *S* = cross-sectional area.

### Antioxidant properties of hydrogels

DPPH solution (40 μg/mL, 4 mL) was added to hydrogel disks (*Ø* 10 mm ×  mm) of three types (GelMA, GelMA–ZC, GelMA–MnPZC) and incubated in the dark at 37°C (pure DPPH as control). After 40 min, the supernatant was collected by high-speed centrifugation, and absorbance was measured at 517 nm (TU-1901 UV–Vis spectrophotometer) with methanol as blank. Scavenging efficiency was calculated using the formula below:


$$\text{DPPH}\;\text{scavenging}\;(\%)=\frac{A_{B}-A_{S}}{A_{B}}\times 100\%$$

*A*_B_: absorbance of DPPH solution, *A*_S_: absorbance of DPPH solution after sample reaction.

### Determination of oxygen release profiles from hydrogels

Hydrogels were prepared into cylinders with a diameter of 15 mm and a height of 10 mm. These cylinders were placed in sample vials containing 10 mL of phosphate buffer. To determine the oxygen release performance of hydrogels at different glucose concentrations and pH levels, hydrogel samples were immersed in different pH buffer solutions containing 3 mg/mL. Oxygen content was measured using a dissolved oxygen meter (JB608, LeiMagnet, Shanghai) at regular intervals. The amount of oxygen produced by the hydrogel during that time period was obtained by subtracting the real time dissolved oxygen value from the measured dissolved oxygen value. Tests were performed for 0.5, 1, 1.5, 2, 3 and 4 days.

### Swelling property test of hydrogel

To evaluate the water absorption properties of hydrogels (GelMA, GelMA–ZC and GelMA–MnPZC), the swelling behavior of different hydrogels was tested. Hydrogel columns (10 mm diameter, 5 mm height) were prepared, and the initial weight (*W*_D_) was measured prior to testing. The hydrogel was then immersed in phosphate buffer and simulated diabetic wound exudate (add 3 mg/mL glucose). Hydrogel weights recorded at different time points were designated as *W*_S_. The swelling ratio (SR) was calculated as follows:


$$\text{SR}\;(\%)=\frac{W_{S}-W_{D}}{W_{D}}\times 100\%$$

### *In vitro* antibacterial properties of hydrogel

*In vitro* antibacterial activity assays were performed using *Escherichia coli* (*E. coli*, gram-negative organism) and *Staphylococcus epidermidis* (*S. epidermidis*, gram-positive organism) to test the antibacterial properties of different hydrogels (GelMA, GelMA–ZC and GelMA–MnPZC). Samples were inoculated with 50 µL of bacterial suspension (1 × 10^6^ CFU mL^−1^). After 4 h, 1 mL of LB broth was added and incubated at 37°C for 12 h. Subsequently, 100 µL was collected for OD600 measurement (MQX200 microplate reader). Antibacterial rates were calculated using the formula below, and plates were then prepared


$$\text{Antibacterial}\;\text{ratio}\;(\%)=\frac{{\text{OD}}_{\text{control}}-{\text{OD}}_{\text{sample}}\;}{{\text{OD}}_{\text{control}}\;}\times 100\%$$

Bacteria were cultured in 6-well plates for 7 days, with the culture medium changed daily to allow biofilm formation. After co-culturing with different hydrogels for 12 h, live/dead bacteria were stained and observed using a laser confocal microscope (CLSM, LSM880, Carl Zeiss, Germany). Quantitative analysis was performed using crystal violet staining.

### Cell activity assay under hypoxic conditions

The promoting effect of hydrogel on cell proliferation and migration was evaluated using rat vascular smooth muscle cells (VSMCs) and mouse fibroblasts (L929).

#### Isolation of VSMCs

A male SD rat (250 g) was euthanized by cervical dislocation, sterilized with 75% alcohol and its thoracic aorta was harvested. After removing the outer and inner vessel layers, the mesentery was retained, minced and transferred to culture flasks. DMEM with 20% FBS was added, and following cell outgrowth, explants were trypsinized (0.25%) and passaged to obtain primary VSMCs.

#### Cell culture

VSMCs (passages 1–8) were cultured in complete medium (10% FBS, 1% penicillin–streptomycin) at 37°C with 5% CO_2_, receiving medium changes every other day.

L929 mouse fibroblasts (Kunming Cell Bank, Kunming, China) were cultured in high-glucose DMEM (Gibco) supplemented with 10% FBS and 1% penicillin–streptomycin at 37°C in a humidified 5% CO_2_ incubator and used for hydrogel cytocompatibility testing.

#### Cell seeding

Different hydrogel samples (GelMA, GelMA–ZC and GelMA–MnPZC) were used for cell viability testing under hypoxic conditions. Hydrogel specimens (*Ø* 8 mm ×  mm) were sterilized with 75% ethanol and washed with sterile PBS/medium. VSMCs (2 × 10^4^ cells/well) were seeded in 24-well plates for 4 h prior to hydrogel addition, then 1 mL medium was added per well, and plates were transferred to a hypoxic chamber (1% O_2_, 37°C). L929 cells (5 × 10^4^ cells/sample) were seeded onto hydrogels in 24-well plates with 1 mL medium per well and cultured in a humidified 5% CO_2_ incubator at 37°C.

#### Live/dead viability

Cells adhered and grew for 1 and 3 days. Biocompatibility and cell proliferation were assessed using live/dead staining analysis and a Cell Proliferation and Viability Assay Kit (CCK-8). Cell morphology was observed using a laser scanning confocal microscope.

#### Cell migration

Three groups of hydrogels (GelMA, GelMA–ZC and GelMA–MnPZC) were used to evaluate their ability to promote cell migration. VSMCs (2 × 10^4^ cells/well) were seeded in 24-well plates and cultured in 1 mL of medium for 48 h, after which hydrogels were added. L929 cells (5 × 10^4^ cells/well) were seeded directly onto hydrogels in 24-well plates, also in 1 mL of medium. After scratching with a 200 μL pipette tip when cell confluence reached 90%, the plates were placed in a hypoxic chamber containing 1% oxygen at 37°C. Live/dead cell staining was performed at 0 and 24 h, and cell migration was observed using a laser scanning confocal microscope.

### *In vitro* reactive oxygen species removal test

RAW264.7 macrophages were seeded in 6-well plates at 1 × 10^7^ cells/well in 1 mL of culture medium. After adherence to the well surfaces, 500 μL of 50 μM hydrogen peroxide was added to each well for stimulation, followed by the addition of hydrogel (GelMA, GelMA–ZC and GelMA–MnPZC). The cells were incubated for a total of 6 h, after which the medium and hydrogel were removed. Cells were washed thrice with PBS, then incubated with DCF-DA (1 mL/well, S0033, Beyotime) for 20 min in the dark, followed by three more PBS washes. Observation of cell morphology and fluorescence was performed using a confocal laser scanning microscope (LSM880, Carl Zeiss).

### *In vitro* immunomodulation test

#### Cell seeding

RAW264.7 macrophages (3 × 10^5^/well) were seeded in 6-well plates (1 mL/well). After adherence, cells were stimulated with LPS (1 μg) and then co-cultured with hydrogels (GelMA, GelMA–ZC or GelMA–MnPZC) under hypoxic conditions (1% O_2_) for 24 h.

#### Immunocytochemical staining

After 24 h co-incubation, cells were washed (2× PBS), fixed (4% paraformaldehyde, 15 min), permeabilized (0.2% Triton X-100, 15 min) and blocked (1% BSA, 1 h). Primary antibodies (rabbit anti-iNOS 1:400, ab283655; mouse anti-CD163 1:400, ab182422; Abcam) were applied overnight at 4°C in the dark. Secondary antibodies (goat anti-rabbit Alexa Fluor 488, 1:1000; donkey anti-rabbit Alexa Fluor 555, 1:800; ab150077, Abcam) were incubated for 2 h at room temperature in the dark. Nuclei were counterstained with DAPI (ab104139). Cells were washed thoroughly with PBS before CLSM observation.

#### RT-qPCR

After 48 h, the expression of macrophage-related genes (IL-6, TNF-α, IL-10, Arg1) in RAW 264.7 cells on different hydrogels was measured by RT-qPCR. Relative quantification was performed using the Δ*Ct* method, with normalization to a housekeeping gene. See Supplementary Table S1 for primer sequences.

### Skin wound repair in diabetic rats

To investigate the diabetic wound-healing efficacy of the hydrogels, a rat model was established in healthy male SD rats (180–220 g, Chengdu Dossy) by intraperitoneal (i.p.) injection of streptozotocin.

Rats were anesthetized with 3% sodium pentobarbital (1 mL/kg). After shaving the back, a GelMA–MnPZC hydrogel (*Ø* 8 mm × 1 mm) was implanted subcutaneously. Swelling was measured periodically, and the degradation rate was determined. On day 10, the skin was incised for degradation observation. Heart, liver, spleen, lung and kidney were collected for H&E staining.

Under anesthesia with 3% sodium pentobarbital (1 mL/kg), diabetic rats were shaved on the back, and four full-thickness wounds (Ø8 mm) were created. Hydrogels were implanted, adhered to the surrounding tissue, and inoculated with 10 μL of mixed bacteria (1 × 10^8^ CFU/mL, *S. epidermidis* and *E. coli*). Wounds were covered with Tegaderm™ dressing (1624W, 3M). To evaluate hydrogel-mediated wound healing, four additional wound treatment groups were established: a control group (bacterial solution only), and GelMA, GelMA-ZC, and GelMA-PZC hydrogel treatment groups. Four replicates were performed in each group. One day after implantation, body fluid and soft tissue from the modeling notch were collected for in vitro bacterial culture. After 4 hours of culture, the culture medium was plated and cultured for another 24 hours to observe colony growth and count the bacteria quantitatively.

Five days post-implantation, rats were euthanized, and wound tissue with surrounding skin was excised. Tissues were fixed in 10% paraformaldehyde, paraffin-embedded, and sectioned. Immunofluorescence staining was performed using anti-4-HNE, HIF-1α, TNF-α, CD86, and CD206 antibodies (sources as indicated). Analysis was conducted to assess oxidative stress and inflammation, thereby evaluating the *in vivo* antioxidant properties of GelMA-MnPZC hydrogels.

At 15 days post-implantation, rats were euthanized, and wound tissues with surrounding skin were excised. Specimens were fixed in 10% paraformaldehyde, paraffin-embedded and sectioned. Histological evaluation of diabetic wound repair by GelMA–MnPZC hydrogels was performed using H&E and Masson’s trichrome staining. Additionally, the effects of the hydrogels on collagen deposition and angiogenesis under varying environmental conditions were assessed by immunofluorescence staining targeting CD31 (ab281583, Abcam), α-SMA (GB111364, Servicebio) and collagen type I (Col I, bs-0578R, Bioss).

All *in vivo* experiments were performed using SPF-grade male SD rats obtained from Chengdu Dossy Experimental Animals Co., Ltd. All procedures were approved by the Medical Ethics Committee of Southwest Jiaotong University (Approval No. SWJTU-2403-NSFC-076) and were conducted in accordance with the Guidelines for the Care and Use of Laboratory Animals (National Research Council, USA).

### Statistical analysis

All data were analysed using one-way analysis of variance (ANOVA) followed by Tukey’s *post hoc* test. Results are presented as mean ± SD, with *n* ≥ 3 per group. Statistical significance was set at *P < 0.05, **P < 0.01, ***P < 0.001, ****P < 0.0001.

## Results and discussion

### Characterization of MnPZC composite nanoparticles

**Figure 2:**
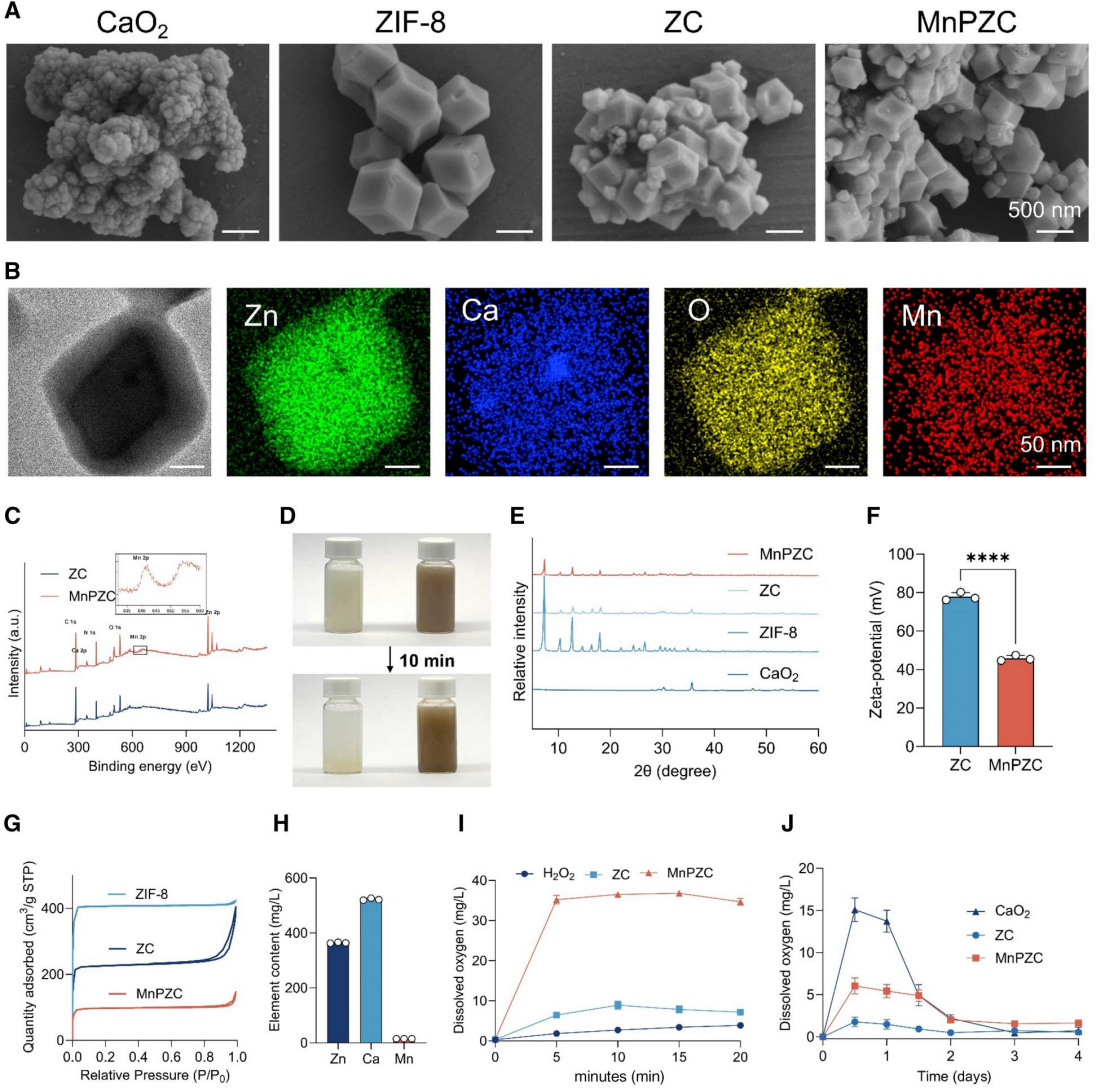
Characterizations of NPs. (**A**) SEM images of CaO_2_, ZIF-8, ZC and MnPZC NPs. (**B**) TEM and EDS elemental mapping images of MnPZC NPs. (**C**) XPS spectra of ZC and MnPZC. (**D**) Visual comparison of the dispersion stability of MnPZC NPs in water. (**E**) XRD analysis confirming the crystallinity of CaO_2_, ZIF-8, ZC and MnPZC NPs. (**F**) Zeta potential analysis of ZC and MnPZC NPs. (**G**) Nitrogen adsorption–desorption isotherms at 77 K for ZIF-8, ZC and MnPZC NPs. (**H**) The content of each element in MnPZC NPs was determined by broad-spectrum atomic absorption spectrometry. (**I**) Catalase activity of ZC and MnPZC NPs. (**J**) Oxygen generation property of CaO_2_, ZC and MnPZC NPs.

Scanning electron microscopy (SEM) images revealed that CaO_2_ consisted of spherical nanoparticles averaging approximately 50 nm in size and exhibiting a clear aggregated state (Figure 2A). In contrast, ZIF-8 MOF particles displayed a regular hexahedral structure with an average size of about 600 nm. The ZC nanoparticles, obtained by *in situ* immobilization of CaO_2_ within ZIF-8, exhibited a less regular hexahedral morphology and a slightly reduced average size of around 450 nm. Following modification with polydopamine (PDA) and Mn^2+^, the resulting MnPZC nanoparticles showed no significant morphological change but exhibited a slight size increase to approximately 500 nm. The distribution of Zn, Ca, O and Mn elements in the MnPZC nanoparticles was confirmed by high-resolution TEM coupled with elemental mapping (HAADF-STEM and EDS) (Figure 2B). The presence of a Mn 2p peak in the XPS spectra of MnPZC indicates successful Mn incorporation (Figure 2C). The successful incorporation of PDA was confirmed by the presence of C=O, C–O and C–N bonds in the high-resolution C 1s spectrum (Figure S1). This result indicates the successful synthesis of MnPZC, with CaO_2_ and Mn^2+^ uniformly distributed inside the ZIF-8 MOF framework. Furthermore, the dispersibility of MnPZC nanoparticles was significantly improved compared to that of bare CaO_2_. After standing for 10 min, CaO_2_ showed significant precipitation, while MnPZC nanoparticles remained well-dispersed (Figure 2D). This improvement is attributed to the catechol groups of PDA, which enhance the diffusion capability of the nanoparticles [32]. X-ray diffraction (XRD) analysis confirmed the good crystallinity of CaO_2_, ZIF-8, ZC and MnPZC nanoparticles. The XRD pattern of MnPZC displayed characteristic peaks from both CaO_2_ and ZIF-8 (Figure 2E), indicating that the introduction of dopamine and Mn^2+^ did not alter the crystalline structure of the nanoparticles. As shown in Figure 2F, the zeta potential of ZC nanoparticles was approximately +77.8 mV, while that of MnPZC nanoparticles increased significantly by about 45.8 mV. The modification of the nanoparticle surface charge is attributed to the amphoteric nature of PDA, which contains amine and phenolic hydroxyl groups. This polymer assumes a negative charge under neutral or alkaline conditions, leading to the observed change [32].

The specific surface areas of the three different nanoparticles were analysed using N_2_ adsorption–desorption experiments (Figure 2G). Compared to ZIF-8, the specific surface area of ZC nanoparticles was significantly reduced to 726 m^2^/g. This decrease is attributed to the partial occupation of the ZIF-8 channels by CaO_2_, leading to a reduction in accessible surface area. The specific surface area of MnPZC nanoparticles decreased further to 418 m^2^/g. This reduction is attributed to the additional occupation of the ZIF-8 channels by the introduced PDA and Mn^2+^, leading to a further decrease in the accessible surface area. The elemental composition of MnPZC was quantified using atomic absorption spectroscopy, which confirmed the release profiles and relative content ratios of Zn, Ca and Mn (Figure 2H). Collectively, these results demonstrate the successful modification of ZC nanoparticles with PDA and Mn^2+^. As shown in Figure 2I, compared with the ZC and H_2_O_2_ control groups, the dissolved oxygen concentration in the MnPZC group reached 34 mg/L within 5 min, confirming O_2_ generation via H_2_O_2_ decomposition. Experimental results show that MnPZC possesses catalase activity. As shown in Figure 2J, the oxygen release profiles of different nanoparticles were evaluated. Compared to bare CaO_2_, both ZC and MnPZC nanoparticles exhibited a more moderate and sustained oxygen release over a period of 4 days. This prolonged release is attributed to the protective effect of the ZIF-8 framework, which enhances the stability of CaO_2_ and prevents a rapid, burst-like oxygen release [16]. Furthermore, the MnPZC nanoparticles showed a significantly higher cumulative oxygen release compared to the ZC nanoparticles. This enhancement is due to the formation of a nanozyme shell with catalase (CAT)-like activity on the surface of ZC nanoparticles following modification with PDA and Mn^2+^. This shell efficiently consumes the H_2_O_2_ generated from the CaO_2_ reaction, thereby boosting O_2_ production via catalytic decomposition while concurrently suppressing the formation of hydroxyl radicals from the non-catalytic decomposition of H_2_O_2_. Overall, the MnPZC nanoparticles demonstrate stable and efficient oxygen-generating performance.

### Physicochemical performance evaluation of GelMA–MnPZC nanocomposite hydrogels

**Figure 3:**
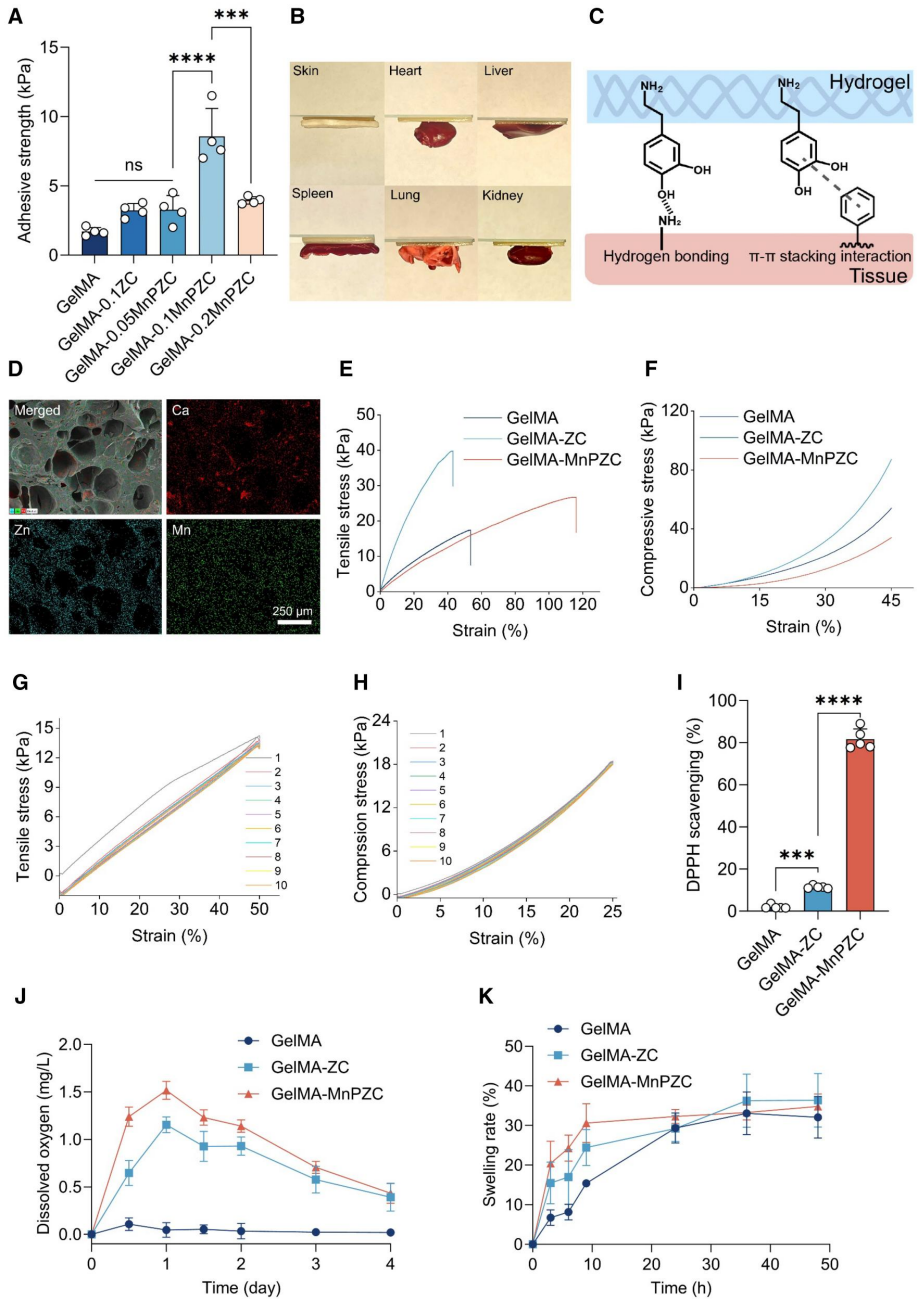
Characterization of the physicochemical properties of hydrogels. (**A**) Comparison of adhesive strengths of GelMA, GelMA–0.1 wt% ZC, GelMA–0.05 wt% MnPZC, GelMA–0.1 wt% MnPZC and GelMA–0.2 wt% MnPZC hydrogels. (**B**) Photographs showing the adhesion of GelMA–MnPZC hydrogel adhesion to different substrates. (**C**) Schematic diagram of hydrogel bioadhesion mechanism. (**D**) SEM images and elemental mapping of the GelMA–MnPZC hydrogel. (**E**) Typical tensile stress–strain curves and (**F**) typical compression stress–strain curves of different hydrogels. Ten cycles of successive loading–unloading tensile curves (**G**) and compression curves (**H**) of the GelMA–MnPZC hydrogel at a strain of 50%. (**I**) DPPH scavenging efficiency of different hydrogels. (**J**) Oxygen release profile of different hydrogels. (**K**) Swelling ratio of the hydrogels in phosphate buffered solution (PBS, pH = 7.4). (*n* ≥ 3, mean ± S.D., n.s. = not significant. *P < 0.05, **P < 0.01, ***P < 0.001, ****P < 0.0001).

The hydrogel exhibits excellent adhesive properties, ensuring stable attachment to wound tissues. Initially, the nanoparticle content within the hydrogel was optimized based on its adhesive performance. As shown in Figure 3A, the GelMA––0.1MnPZC hydrogel displayed higher adhesive strength compared to the GelMA and GelMA–0.1ZC groups, which is attributed to the enhanced interfacial bonding capability imparted by the incorporated PDA. The adhesive strength of the hydrogel initially increased and then decreased with rising nanoparticle concentration, reaching a maximum value of approximately 8.6 kPa at an MnPZC content of 0.1%. This trend results from the coagulation effect induced by higher nanoparticle loading, which compromises the hydrogel’s cohesive and adhesive integrity. The hydrogel demonstrated stable adhesion to various biological surfaces, including skin and internal organs (Figure 3B). Drawing inspiration from mussel adhesion, the hydrogel achieves robust binding to various substrates through its PDA-provided phenolic hydroxyl groups, which engage in multiple molecular interactions, including hydrogen bonding, coordination bonds, covalent linkages, cation–π interactions and π–π stacking (Figure 3C) [33]. SEM characterization revealed that all hydrogel groups retained a porous architecture, and the incorporation of nanoparticles did not alter their intrinsic structural morphology (Figure S2). In contrast, the GelMA–MnPZC hydrogel exhibited more uniform dispersion of nanoparticles on its surface. Energy-dispersive X-ray spectroscopy (EDS) mapping confirmed the incorporation of MnPZC within the hydrogel, showing homogeneous distribution of Zn and Mn signals throughout the porous matrix (Figure 3D). This improved dispersion stems from the abundant catechol groups in PDA, which readily interact with metal ions on the ZIF-8 framework, forming a PDA coating that enhances nanoparticle compatibility and distribution within the hydrogel network. The resulting interconnected porous structure supports cellular infiltration, tissue integration and efficient nutrient transport.

The hydrogel exhibits excellent mechanical properties, allowing the dressing to conform perfectly to skin tissue. The mechanical performance of the hydrogel was evaluated through compression and tensile tests (Figure 3E and F). As shown in Supplementary Figure S3A and D, under 40% strain, the GelMA–ZC hydrogel demonstrated higher tensile strength (35 kPa) and compressive strength (88 kPa) compared to the pure GelMA hydrogel, which can be attributed to the reinforcement effect provided by the incorporated nanoparticles within the hydrogel network. In contrast, the GelMA–MnPZC hydrogel exhibited a decrease in both tensile strength (26 kPa) and compressive strength (40 kPa). A similar trend was observed in the elastic modulus (Supplementary Figure S3B and E). This reduction in mechanical properties is likely due to the phenolic hydroxyl groups of PDA consuming a portion of the free radicals during thermal polymerization, thereby partially hindering the cross-linking of GelMA and leading to a less dense network [19, 34]. Notably, the stretch ratio of the GelMA–MnPZC hydrogel increased significantly, reaching 200% (Supplementary Figure S3C), indicating enhanced flexibility and improved adaptability to dynamic skin tissue. Furthermore, cyclic tensile and compression tests performed on the GelMA–MnPZC hydrogel revealed consistent load––unloading hysteresis loops over 10 consecutive cycles (Figure 3G and H), with no evidence of bond breakage or permanent structural damage after repeated loading. These results demonstrate the excellent deformation recovery and fatigue resistance of the GelMA–MnPZC hydrogel, which is essential for withstanding the cyclic mechanical stresses encountered during daily skin movement and tissue repair.

As demonstrated in Figure 3I and Supplementary Figure S4, the GelMA–MnPZC hydrogel shows a high DPPH free-radical scavenging efficiency, confirming its potent antioxidant capacity. This property enables the hydrogel to effectively mitigate oxidative stress in diabetic wounds, thereby establishing a microenvironment that supports tissue regeneration. Moreover, the GelMA–MnPZC hydrogel exhibits a sustained self-oxygenation capability, releasing oxygen continuously and stably over a period of up to 4 days (Figure 3J). The cumulative oxygen release from the GelMA–MnPZC hydrogel is significantly greater than that of the GelMA–ZC hydrogel, indicating that the incorporation of PDA-Mn nanozymes substantially enhances the oxygen-generation efficiency of the system. As shown in Supplementary Figure S5A, pH had a certain impact on the oxygen release capacity of the hydrogel. In an acidic environment with pH = 6, the oxygen release was higher than that in the neutral and alkaline experimental groups on the first day, but gradually stabilized over time. This may be because the acidic pH environment promoted the degradation of ZIF-8 [32], and the rapid release of CaO_2_ led to an increase in oxygen release in the early stage. The results indicate that the hydrogel is more adapted to the acidic microenvironment of diabetic wounds, rapidly releasing oxygen to improve inflammation and promote angiogenesis. Glucose concentration did not have a significant effect on the oxygen release behavior of the hydrogel (Supplementary Figure S5B). In addition, the hydrogel displays a low swelling ratio in phosphate buffer (Figure 3K) and simulated diabetic wound exudate (Supplementary Figure S6), which contributes to the stable adhesion of the dressing at the wound site and helps maintain a consistent interface during the healing process. The hydrogel significantly decreased in size 5 and 10 days after implantation in rats (Supplementary Figure S7A). Quantitative analysis of the degradation curves showed that the hydrogel degraded, and by day 10 it was almost completely degraded (Supplementary Figure S7B). This indicates that the hydrogel exhibits good degradation performance *in vivo*.

### Antibacterial properties of GelMA–MnPZC hydrogel

**Figure 4:**
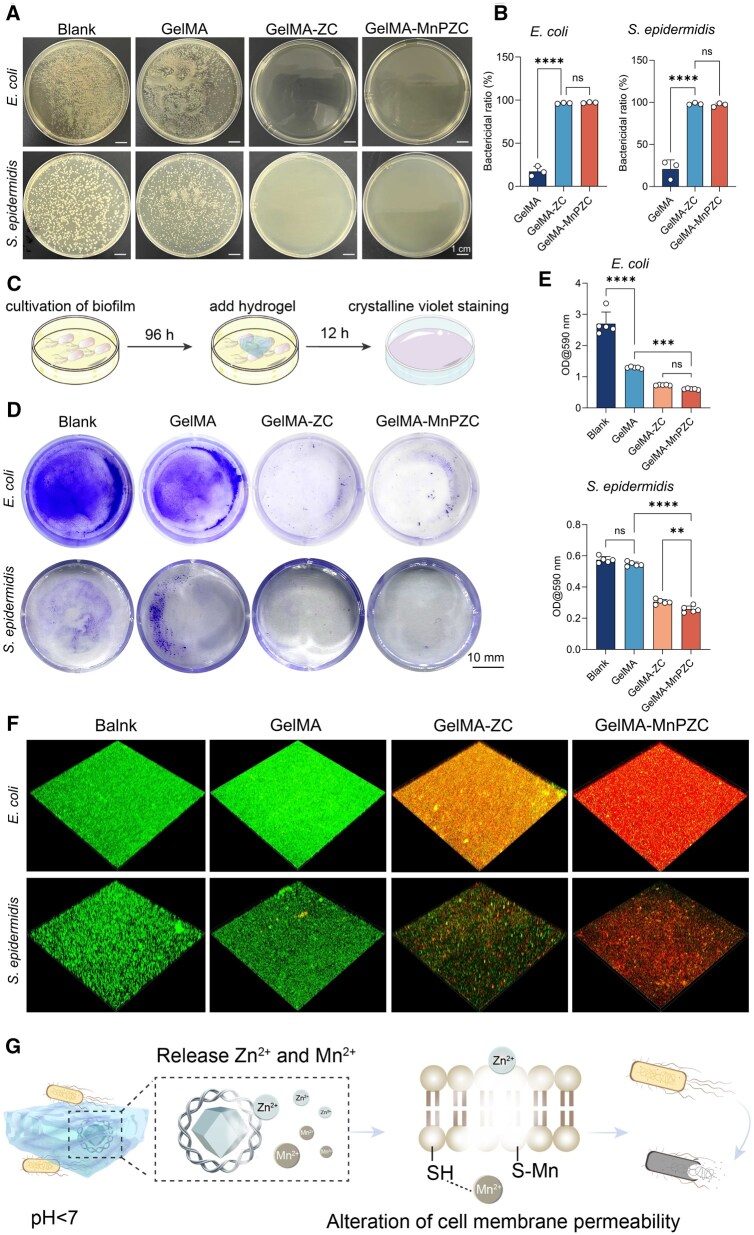
In vitro antibacterial properties of hydrogels. (**A**) Agar plate photographs of *E. coli* and *S. epidermidis* after 12 h co-culture with various hydrogels (37°C). (**B**) Quantified antibacterial rates of the tested hydrogels. (**C**) Schematic diagram of the experimental procedure for evaluating the antibacterial properties of hydrogels. (**D**) Representative images of crystal violet-stained *E. coli* and *S. epidermidis* biofilms following 12 h co-culture with various hydrogels. (**E**) Quantitative analysis of biofilm biomass measured as crystal violet absorbance at 590 nm. (**F**) Three-dimensional CLSM micrographs of biofilms labeled with Calcein-AM (live cells, green) and PI (dead cells, red) after different treatments. (**G**) Schematic diagram of the antibacterial mechanism of GelMA–MnPZC hydrogel. (*n* ≥ 3, mean ± S.D., n.s. = not significant. *P < 0.05, **P < 0.01, ***P < 0.001, ****P < 0.0001).

The GelMA–MnPZC hydrogel demonstrated significant antibacterial properties, which enhance its ability to protect wounds from bacterial infection. The antibacterial activity of the material was assessed using *E. coli* (a representative gram-negative bacterium) and *S. epidermidis* (a gram-positive bacterium) as test strains. As shown in Figure 4A and B, no visible *E. coli* and *S. epidermidis* colonies were observed on agar plates treated with GelMA–ZC or GelMA–MnPZC hydrogels. Quantitative analysis revealed that after 12 h of co-incubation, the GelMA–MnPZC hydrogel achieved an antibacterial ratio exceeding 97% against *E. coli* and 95% against *S. epidermidis*, confirming its highly efficient bactericidal performance. The inhibition of biofilm formation of the GelMA–MnPZC hydrogel was further evaluated using *E. coli* and *S. epidermidis* biofilm model (Figure 4C). Biofilms were stained with crystal violet and quantified by measuring the absorbance at 590 nm. As shown in Figure 4D and E, the GelMA–MnPZC treatment group exhibited the lowest staining intensity among all groups, indicating effective suppression of biofilm formation. Additionally, live/dead staining combined with confocal laser scanning microscopy (CLSM) was performed (Figure 4F). Biofilms in the blank and GelMA control groups displayed dense, intact structures with strong green fluorescence, indicating viable bacteria. In the GelMA–ZC group, red fluorescence corresponding to dead bacteria was observed, suggesting partial biofilm disruption. In contrast, the GelMA–MnPZC group showed predominantly red fluorescence, indicating that nearly all bacteria within the biofilm were non-viable. The *in vivo* antibacterial experiments yielded results that were consistent with those from *in vitro* tests (Supplementary Figure S8). These results suggest that the GelMA–MnPZC hydrogel exerts its antibacterial effect through multiple mechanisms. First, it may electrostatically adsorb to bacterial cell membranes, compromising membrane integrity and inducing leakage of cellular contents [35]. Second, the released Zn^2+^ and Mn^2+^ ions can chelate with components of the bacterial outer membrane, altering cell-wall permeability and disrupting bacterial structure [36]. Third, by binding to essential bacterial macromolecules such as proteins, enzymes and DNA, the polyphenol groups in the hydrogel inhibit metabolic activity and interfere with normal physiological functions [37]. The synergistic action of divalent metal ions and PDA endows the GelMA–MnPZC hydrogel with potent antibacterial and anti-biofilm activity, highlighting its promising potential for the treatment of infected wounds.

### GelMA–MnPZC hydrogel enhanced cell proliferation and migration under hypoxic conditions

**Figure 5:**
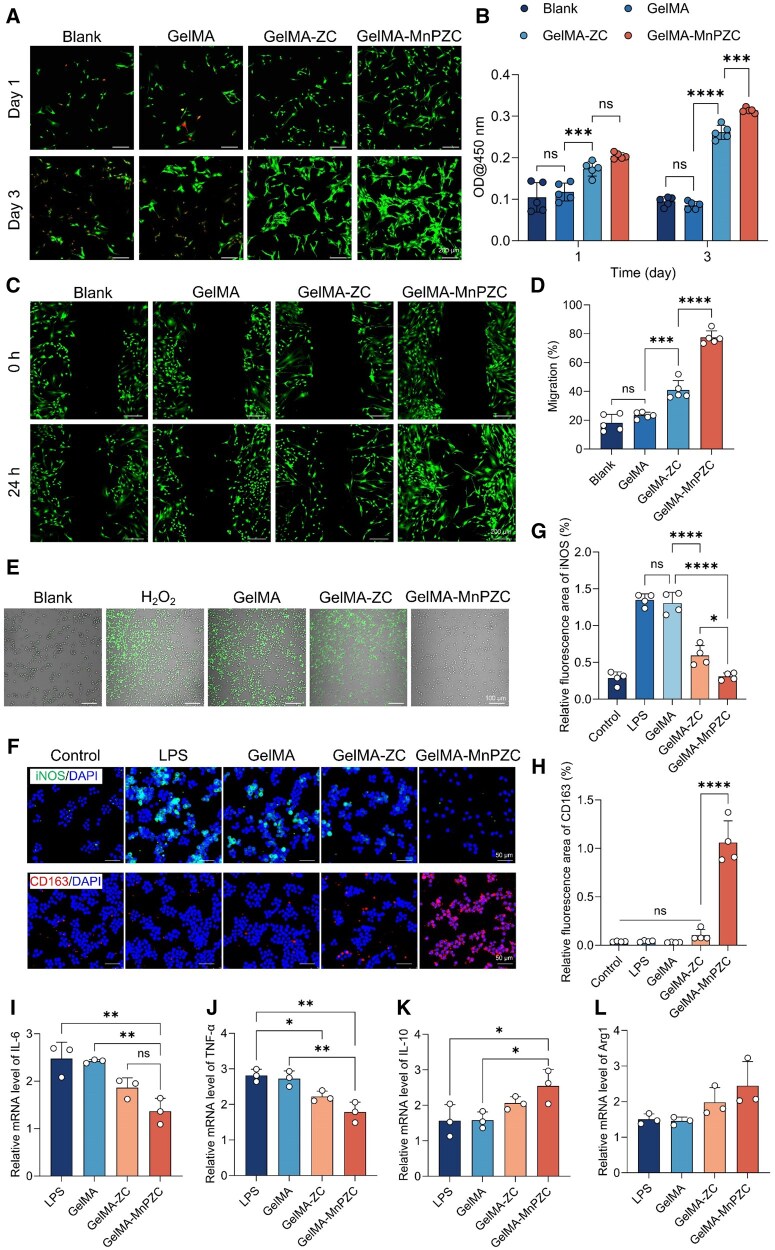
Cytocompatibility, *in vitro* antioxidant and anti-inflammatory properties of GelMA–MnPZC hydrogel. (**A**) Representative live/dead staining images and (**B**) proliferation of VSMC cell on different hydrogels in a hypoxic environment for 1 and 3 days. (**C**) Representative phase-contrast images and (**D**) quantitative analysis of VSMC cells migration to the scratched area on different hydrogels. (**E**) Evaluation of intracellular ROS-scavenging activity of different hydrogels. (**F–H**) Representative immunofluorescence images and corresponding quantitative analysis of iNOS (M1 marker) and CD163 (M2 marker) expression following 1 day of co-culture with various hydrogels. (**I, G, K, L**) qPCR analysis of pro-inflammatory (iNOS, IL-6) and anti-inflammatory (IL-10, Arg1) mRNA levels in LPS-stimulated macrophages treated with different hydrogels. (*n* ≥ 3, mean ± S.D., n.s. = not significant. *P < 0.05, **P < 0.01, ***P < 0.001, ****P < 0.0001).

The self-oxygenation capability of the GelMA–MnPZC hydrogel effectively promoted cellular activity under hypoxic conditions. VSMCs and L929 cells were co-cultured with various hydrogels in a low-oxygen environment (1% O_2_). Evaluation using CCK-8 assays (Figure 5B and Supplementary Figure S10B) and confocal microscopy imaging after 1 and 3 days of co-culture (Figure 5A and Supplementary Figure S10A) showed that both GelMA–ZC and GelMA–MnPZC hydrogels significantly enhanced VSMCs and L929 cells proliferation under hypoxia. In contrast, the GelMA-only hydrogel failed to support VSMCs and L929 cells survival and proliferation under the same conditions. A consistent trend was observed for HaCaT cells after 3 days of co-culture (Supplementary Figure S9), confirming that the lack of oxygen-releasing capacity in the control groups led to impaired cell viability. Notably, the proliferation rate of VSMCs in the GelMA–MnPZC group was higher than that in the GelMA–ZC group, which can be attributed to the improved oxygen-generation efficiency resulting from the incorporation of the PDA-Mn nanozyme. Furthermore, cell migration under hypoxia was examined using a scratch assay. Confocal images (Figure 5C and Supplementary Figure S10C) and quantitative analysis (Figure 5D and Supplementary Figure S10D) of VSMCs and L929 cells at 0 and 24 h post-scratch demonstrated that the GelMA–MnPZC hydrogel significantly promoted cell migration and accelerated gap closure under hypoxic conditions. We attribute this enhancement to the hydrogel’s sustained oxygen release, which facilitates the outgrowth of cellular protrusions (including lamellipodia and filopodia). Consequently, cell spreading, proliferation and motility are promoted, all of which positively influence the wound healing process.

### Antioxidant and inflammatory moderating capabilities of GelMA–MnPZC hydrogel

The GelMA–MnPZC hydrogel exhibits significant antioxidant properties, enabling it to scavenge ROS and thereby protect cells from oxidative damage. To evaluate its intracellular ROS-scavenging ability, RAW 264.7 macrophages were co-cultured with the hydrogel. Confocal microscopy images (Figure 5E) revealed that after stimulation with 50 μM H_2_O_2_, cells cultured with GelMA or GelMA–ZC hydrogels showed a marked increase in intracellular ROS, indicated by strong green fluorescence. In contrast, the GelMA–MnPZC group displayed significantly lower ROS levels. The excellent ROS-scavenging capacity of the GelMA–MnPZC hydrogel, as demonstrated by these results, arises from the abundant reducing phenolic hydroxyl groups present in MnPZC. These groups act as effective electron donors and exhibit strong radical-interacting activity [38]. In addition to the antioxidant capacity of PDA, the presence of Mn^2+^ in the hydrogel may also contribute to ROS scavenging. Mn^2+^ is known to possess SOD-mimetic activity, catalysing the dismutation of superoxide radicals [39]. Therefore, the overall antioxidant property of the hydrogel is likely attributable to the combined or synergistic effects of PDA and Mn^2+^.

Furthermore, the GelMA–MnPZC hydrogel shows notable immunomodulatory effects under hypoxic conditions. To investigate this, RAW 264.7 macrophages were stimulated with lipopolysaccharide (LPS) and cultured on different hydrogels for 48 h. Immunofluorescence staining was used to identify M1 and M2 macrophage phenotypes via markers iNOS (M1) and CD163 (M2), respectively (Figure 5F). The GelMA–MnPZC treated group exhibited fewer iNOS-positive M1 macrophages and a significantly higher number of CD163-positive M2 macrophages (Figure 5G and H). The polarization effect was further confirmed by qPCR analysis of pro-inflammatory (IL-6, TNF-α) and anti-inflammatory (IL-10, Arg1) gene expression (Figure 5I–L). Macrophages treated with GelMA–MnPZC hydrogel showed downregulated expression of IL-6 and TNF-α, alongside upregulated expression of IL-10 and Arg1. A direct link between antioxidant activity and immunomodulation is established by the PDA within the hydrogels, which both scavenges ROS and promotes macrophage polarization from the pro-inflammatory M1 to the anti-inflammatory M2 phenotype [19, 40]. Antioxidant activity translates into immunomodulation via NF-κB and STAT6 signaling. Excessive ROS activates the NF-κB pathway, driving transcription of pro-inflammatory cytokines including TNF-α and IL-6. Conversely, the anti-inflammatory response is orchestrated by STAT6 signaling, which promotes expression of M2 macrophage polarization associated markers IL-10 and Arg1. We reason that the hydrogel likely exerts its immunomodulatory effects through NF-κB suppression and STAT6 activation [41, 42]. Collectively, these findings indicate that the GelMA–MnPZC hydrogel not only mitigates oxidative stress but also alleviates excessive inflammation by promoting macrophage polarization toward an anti-inflammatory M2 phenotype. This dual function helps establish a favorable microenvironment conducive to accelerated wound repair.

### GelMA–MnPZC hydrogel promotes wound healing in diabetic rats

**Figure 6:**
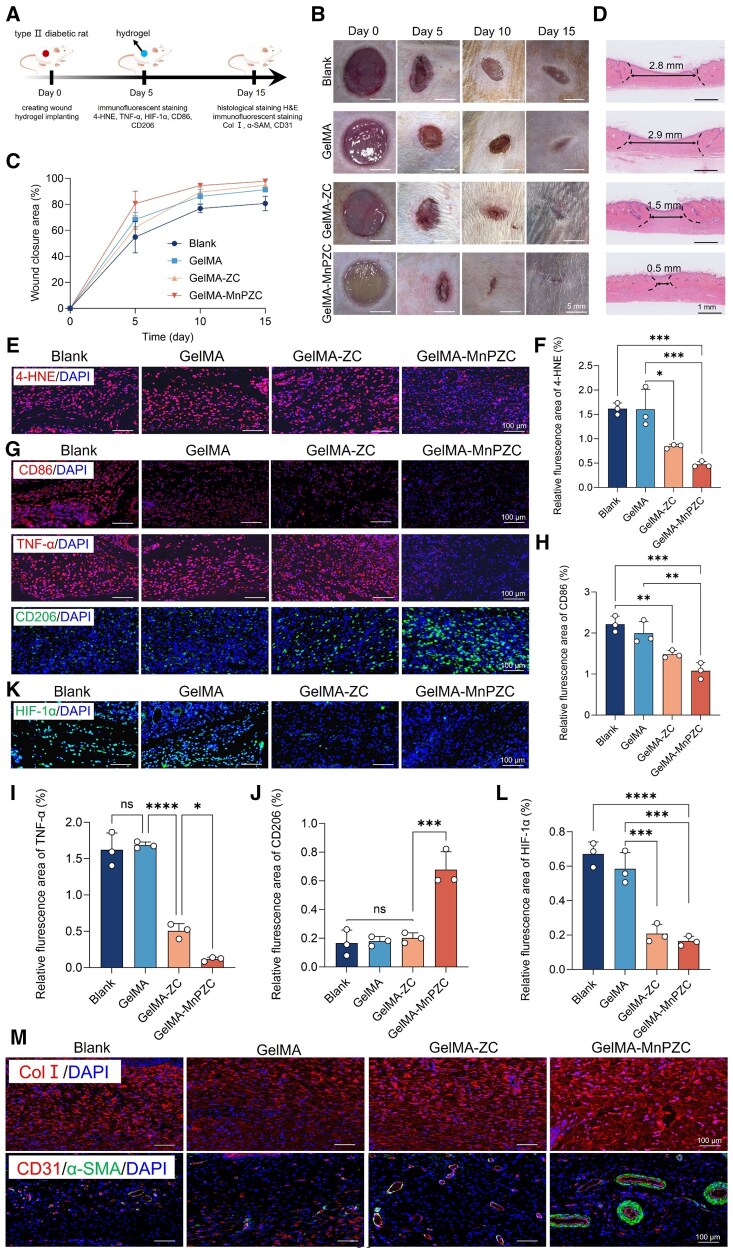
GelMA–MnPZC hydrogel accelerating wound healing and regeneration in a diabetic rat. (**A**) Schematic depicting the workflow used to assess diabetic wound healing. (**B**) Time-course digital images of wound healing across all experimental groups. (**C**) Quantified wound closure rates for each group during the healing period. (**D**) Representative H&E histology of wound sites on day 15 for the indicated groups. (**E**) Day 5 wound immunofluorescence: 4-HNE (oxidative stress marker) staining. (**F**) Quantified 4-HNE fluorescence intensity. (**G**) Day 5 immunofluorescence for CD86, iNOS and CD206. (**H–J**) Corresponding fluorescence quantification. (**K**) Day 5 HIF-1α staining in wounds. (**L**) Quantified HIF-1α intensity. (**M**) Day 15 immunofluorescence for α-SMA, CD31 and Col I; DAPI for nuclei. (*n* ≥ 3, mean ± S.D., n.s. = not significant. *P < 0.05, **P < 0.01, ***P < 0.001, ****P < 0.0001).

GelMA–MnPZC hydrogel effectively alleviates local tissue hypoxia, modulates the inflammatory microenvironment and accelerates wound healing in diabetic conditions. Impaired glucose metabolism and compromised vascular oxygen delivery in diabetic wounds lead to hypoxia and a pro-inflammatory milieu. Together, these factors suppress angiogenesis, re-epithelialization and extracellular matrix synthesis, thereby hindering wound healing [43]. A full-thickness skin defect model was created in diabetic rats to assess the wound dressing potential of the hydrogel (Figure 6A). The GelMA–MnPZC group exhibited a significantly higher wound healing rate compared with all other groups (Figure 6C). Moreover, as shown in Figure 6B, following 5 days of treatment, the wound area in the GelMA–MnPZC group was markedly reduced relative to the blank, GelMA, and GelMA–ZC groups. This healing advantage became more pronounced at day 10. By day 15, wounds treated with GelMA–MnPZC hydrogel were nearly completely closed, whereas the other groups exhibited varying degrees of delayed healing. Among the controls, the GelMA–ZC group showed slightly better healing compared to the blank and GelMA-only groups. Histological analysis via H&E staining (Figure 6D) and Masson’s trichrome staining (Supplementary Figure S11) further revealed that the newly formed tissue in the GelMA–MnPZC group exhibited higher structural maturity, a significantly reduced granulation tissue width (only 0.5 mm), and the presence of hair follicles. These findings indicate that the GelMA–MnPZC hydrogel not only accelerates diabetic wound closure but also promotes more organized and physiologically functional tissue regeneration.

To elucidate the mechanism by which the hydrogel promotes diabetic wound repair, we performed immunofluorescence staining on wound tissue sections. The protective effect of the hydrogel against oxidative stress was first evaluated by detecting 4-hydroxy-2-nonenal (4-HNE), a well-established marker of lipid peroxidation [44]. The results showed that 4-HNE levels in the GelMA–ZC and GelMA–MnPZC groups were significantly lower than those in the blank and GelMA-only groups, with the GelMA–MnPZC group exhibiting the lowest signal intensity (Figure 6E and F). This indicates that the GelMA–MnPZC hydrogel effectively mitigates oxidative stress in wound tissue through its antioxidant activity. These results indicate that the sustained oxygen release and polyphenol-mediated antioxidant properties of the hydrogel act synergistically to suppress tissue oxidative damage. We further investigated the anti-inflammatory properties of the hydrogel by analysing macrophage infiltration and polarization in the wound bed. Immunofluorescence staining was performed using CD86 and TNF-α (M1) and CD206 (M2) as specific markers. As shown in Figure 6G, strong red fluorescence was observed in the blank and GelMA groups, indicative of a pronounced inflammatory response. In contrast, the GelMA–ZC and GelMA–MnPZC groups showed markedly reduced red fluorescence, with the GelMA–MnPZC group displaying the weakest signal. Conversely, green fluorescence corresponding to CD206-positive M2 macrophages was most intense in the GelMA–MnPZC group. Quantitative analysis revealed that the expression of pro-inflammatory markers (CD86 and TNF-α) followed the order: blank ≈ GelMA > GelMA–ZC > GelMA–MnPZC (Figure 6H and I), while the anti-inflammatory marker CD206 showed the opposite trend: GelMA–MnPZC > GelMA–ZC > GelMA ≈ Blank (Figure 6J). These *in vivo* results align with the *in vitro* cellular data, demonstrating that the sustained oxygen release from the GelMA–MnPZC hydrogel, combined with the anti-inflammatory properties of PDA, promotes macrophage polarization from the pro-inflammatory M1 phenotype toward the anti-inflammatory M2 phenotype. This immunomodulatory effect helps reshape the wound immune microenvironment into one that favors resolution of inflammation and supports tissue regeneration.

The ability of the GelMA–MnPZC hydrogel to release oxygen is critical for promoting angiogenesis and tissue regeneration. To evaluate the effect of hydrogel-derived oxygen on vascularization, wound tissue sections collected after 5 days of treatment were immunostained for hypoxia-inducible factor-1α (HIF-1α) (Figure 6K) [45]. Additionally, tissues harvested on day 15 were stained for the endothelial marker CD31 and the pericyte/smooth muscle marker α-SMA to assess mature blood vessel formation (Figure 6M) [46, 47]. As shown in Figure 6L, the immunofluorescence intensity of HIF-1α was lowest in the GelMA–MnPZC group, indicating effective mitigation of tissue hypoxia. Correspondingly, on day 15, both CD31 and α-SMA signals were significantly stronger in the GelMA–MnPZC group than in all other groups (Supplementary Figure S12A and B). These results demonstrate that the sustained oxygen release from the hydrogel enhances oxygen availability in the wound bed, supports aerobic metabolism and stimulates robust angiogenesis. The newly formed vasculature is essential for delivering nutrients and oxygen to support the repair process [48]. Finally, immunofluorescence staining for collagen I was performed to evaluate collagen deposition in the regenerated tissue. The GelMA–MnPZC group exhibited more abundant and well-organized collagen I fibers, indicating that the hydrogel promotes structural remodeling and functional restoration of the injured skin. In summary, the GelMA–MnPZC hydrogel orchestrates diabetic wound healing through a coordinated multi-stage process: (1) antibacterial action via released divalent ions; (2) modulation of the hypoxic and inflammatory microenvironment through sustained oxygen release and polyphenol-mediated antioxidant/anti-inflammatory effects; (3) promotion of angiogenesis and tissue regeneration and (4) enhancement of collagen deposition and extracellular matrix remodeling. Collectively, these functions facilitate rapid, high-quality healing of diabetic wounds. As shown in Supplementary Figure S13, 10 days after hydrogel implantation, H&E sections of major organs showed no significant difference compared to the blank control group, indicating that the hydrogel has good long-term biocompatibility. Therefore, the GelMA–MnPZC hydrogel dressing offers a promising approach for diabetic wound management, holding substantial potential for clinical translation.

## Conclusion

This study addresses two critical challenges in diabetic wound healing: hypoxia and excessive inflammation. We have developed a stable, self-oxygenating hydrogel dressing mediated by a polyphenol–metal nanozyme system. This dressing remodels the hostile wound microenvironment through three coordinated actions: antibacterial divalent ion release, polyphenol-driven antioxidant activity and sustained, efficient oxygen supply, thereby promoting high-quality and accelerated tissue repair. The system employs a ZIF-8-encapsulated CaO_2_ core combined with a PDA-Mn nanozyme shell, which significantly improves the reactivity stability and oxygen-generation efficiency of CaO_2_. Compared with conventional oxygen-carrying dressings, this hydrogel provides more stable and efficient oxygen release, while the incorporated polyphenols confer durable antioxidant and immunomodulatory functions. Furthermore, the catechol groups from polyphenols enhance cell adhesion, proliferation and migration, contributing to faster wound closure. A polyphenol-mediated anchoring strategy also enables uniform dispersion of the MnPZC composite nanoparticles within the hydrogel network, improving its mechanical properties and tissue adhesion. As a result, the hydrogel can adhere persistently to the wound bed, continuously releasing oxygen and bioactive agents to optimize the healing microenvironment, enabling comprehensive management of diabetic wounds. In summary, this polyphenol-nanozyme-augmented self-oxygenating hydrogel offers a promising strategy for microenvironment remodeling and accelerated repair of diabetic wounds.

## Supplementary Material

rbag118_Supplementary_Data
