# Supplementary material for: A self-oxygenating polyphenol-nanozyme hydrogel remodels the inflammatory microenvironment for diabetic wound healing
Source: Regen Biomater. 2026 Jun 12;13:rbag118. doi: 10.1093/rb/rbag118 (PMC13355597; doi:10.1093/rb/rbag118)
Supplement: rbag118_Supplementary_Data [file rbag118_supplementary_data.docx]

Supporting Information

**A self-oxygenating polyphenol-nanozyme hydrogel remodels the inflammatory microenvironment for diabetic wound healing**

*Shangqing Huang^a b c^, Yujia Zheng^a^, Shengxi Jiang^a^, Huabin Liu^a^, Lijun Tang^a b d*^, Chaoming Xie^a b *^, Jie Weng ^a b*^ and Yanan Jiang^e f *^*

a. Key Laboratory of Advanced Technologies of Materials Ministry of Education, Institute of Biomedical Engineering, College of Medicine, Southwest Jiaotong University, Chengdu, Sichuan, 610031 China

b. School of Materials Science and Engineering, Southwest Jiaotong University, Chengdu, 610031 China

c. Department of Gastroenterology, Chengdu Integrated TCM & Western Medicine Hospital, Chengdu, Sichuan Province, 610059, China.

d. Tissue Stress Injury and Functional Repair Key Laboratory of Sichuan Province，The General Hospital of Western Theater Command (Chengdu Military General Hospital), Chengdu 610083, Sichuan Province, China

e. Obesity and Metabolism Medicine-Engineering Integration Laboratory, Department of General Surgery, The Affiliated Hospital of Southwest Jiaotong University, The Third People's Hospital of Chengdu, Chengdu, 610031, China

f. Medical Research Center, The Third People's Hospital of Chengdu, Affiliated Hospital of Southwest Jiaotong University, Chengdu, China

^*^ Corresponding author.

*E-mail addresses:* 596041159@qq.com (YN. Jiang), jweng@swjtu.edu.cn (J. Weng), xie@swjtu.edu.cn (CM. Xie), tanglj2016@163.com (LJ. Tang)

**Table S1. Primer sequences for RT-qPCR**

| Gene |  | Sequence (5'-3') | Amplicon size (bp) |
| --- | --- | --- | --- |
| GAPDH | forward | GGTTGTCTCCTGCGACTTCA | 183 |
|  | reverse | TGGTCCAGGGTTTCTTACTCC |  |
| IL-6 | forward | AGTTCCTCTCTGCAAGAGACTTCC | 109 |
|  | reverse | TTGCCATTGCACAACTCTTTTC |  |
| TNF-α | forward | TCAAAATTCGAGTGACAAGCCTG | 245 |
|  | reverse | GGTATGAGATAGCAAATCGGCTG |  |
| IL-10 | forward | CAACATACTGCTAACCGACTC | 77 |
|  | reverse | GGATCATTTCCGATAAGG |  |
| Arg1 | forward | CTTGCGAGACGTAGACC | 102 |
|  | reverse | ATCACCTTGCCAATCCC |  |


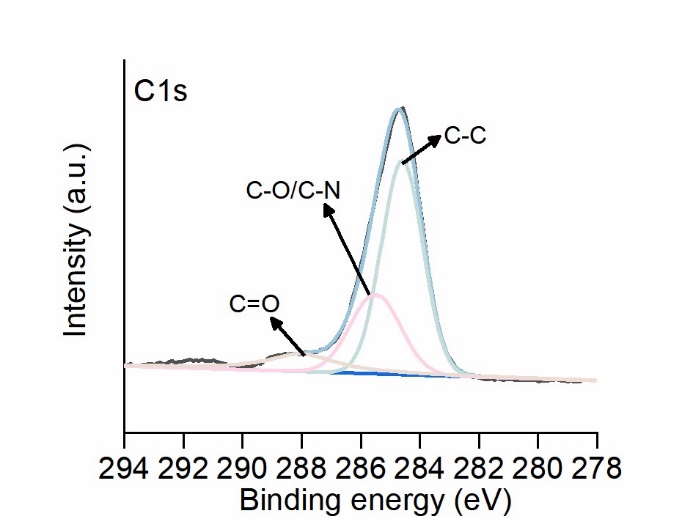


Fig. S1. High resolution XPS spectra of C 1 s of MnPZC.


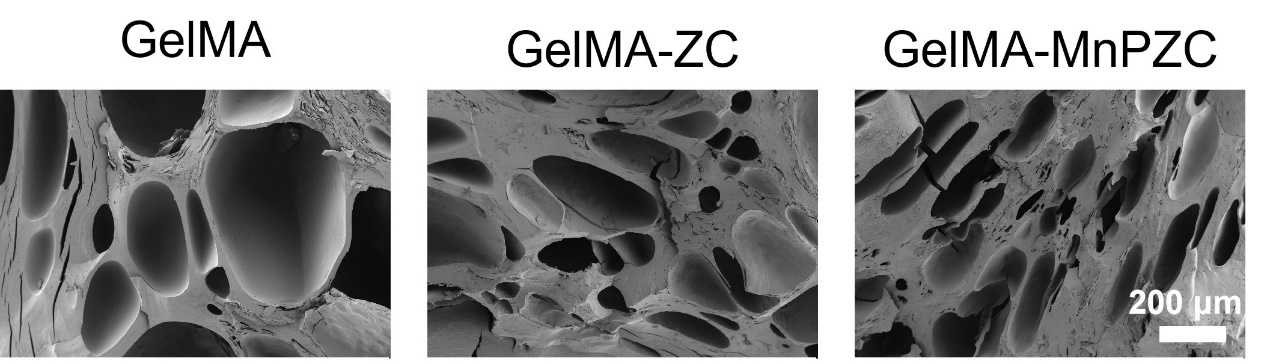


Fig. S2. SEM images of different hydrogels.


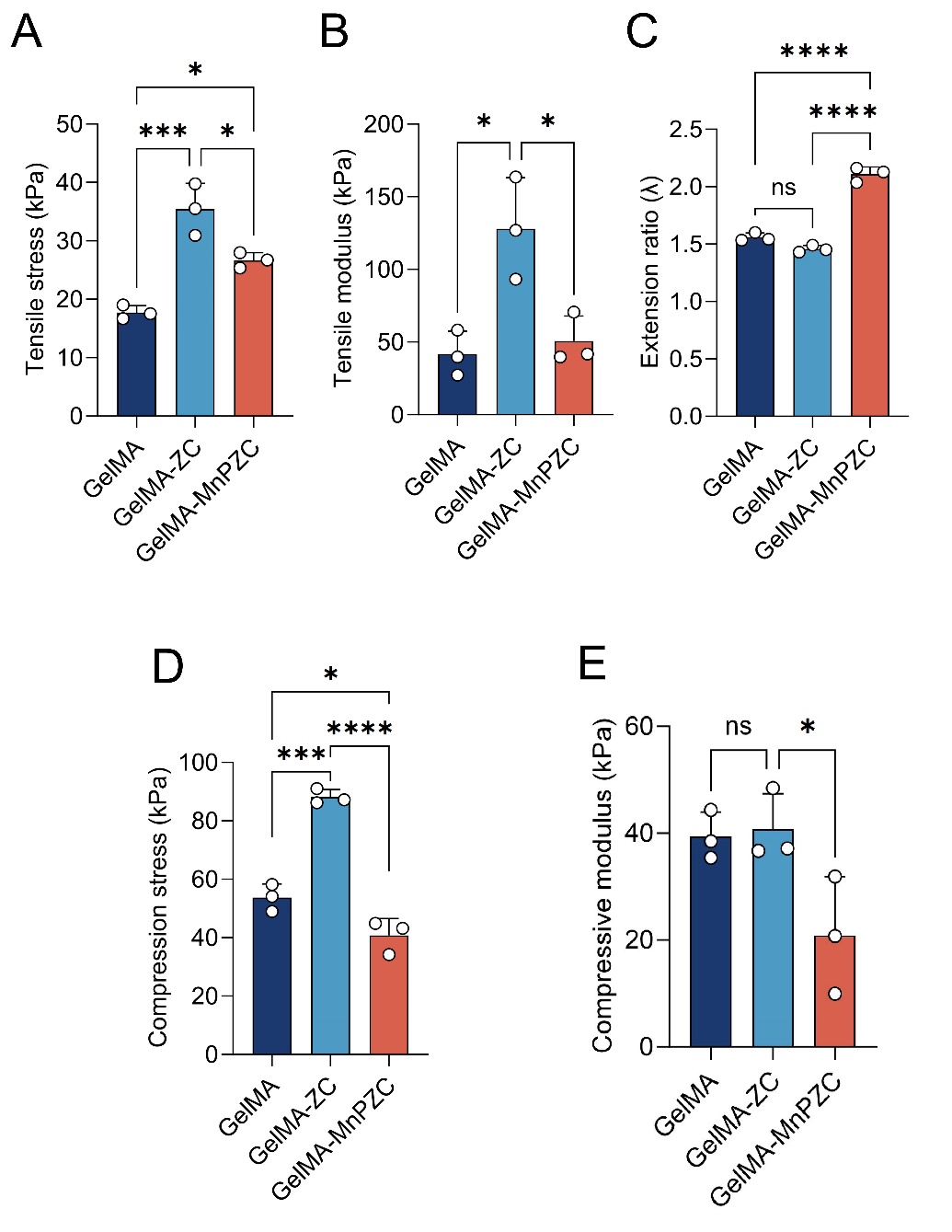


Fig. S3. Tensile strength(A), moduli (B), and extension ratio (C) of GelMA, GelMA-ZC and GelMA-MnPZC hydrogels. (D) Compression strengths of different hydrogels. (E) Compression modulus of different groups of hydrogels.


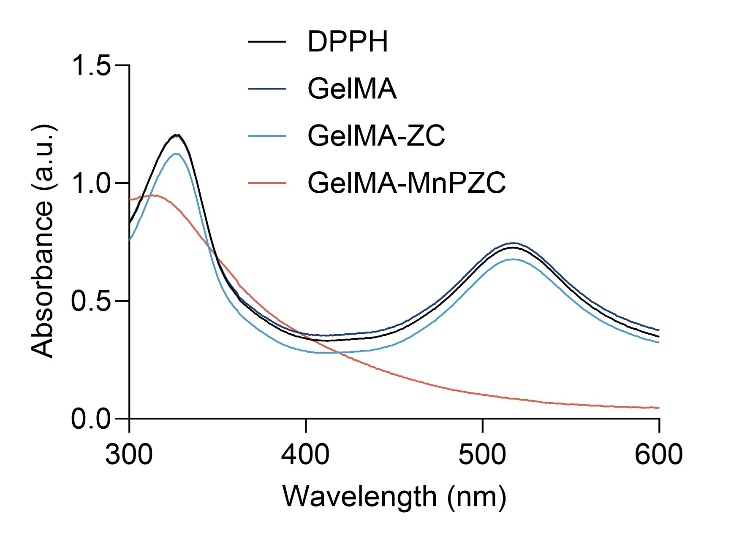


Fig. S4. UV spectra of various hydrogels analyzed after a 90-min reaction with DPPH free radicals.


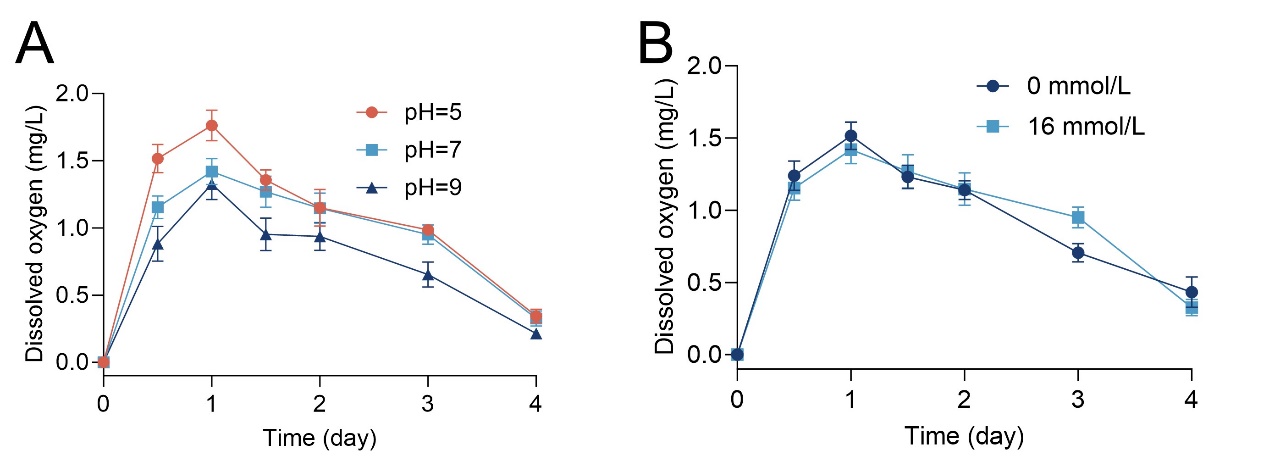


Fig. S5. Oxygen release behavior of hydrogels at different pH (A) and glucose concentrations (B).


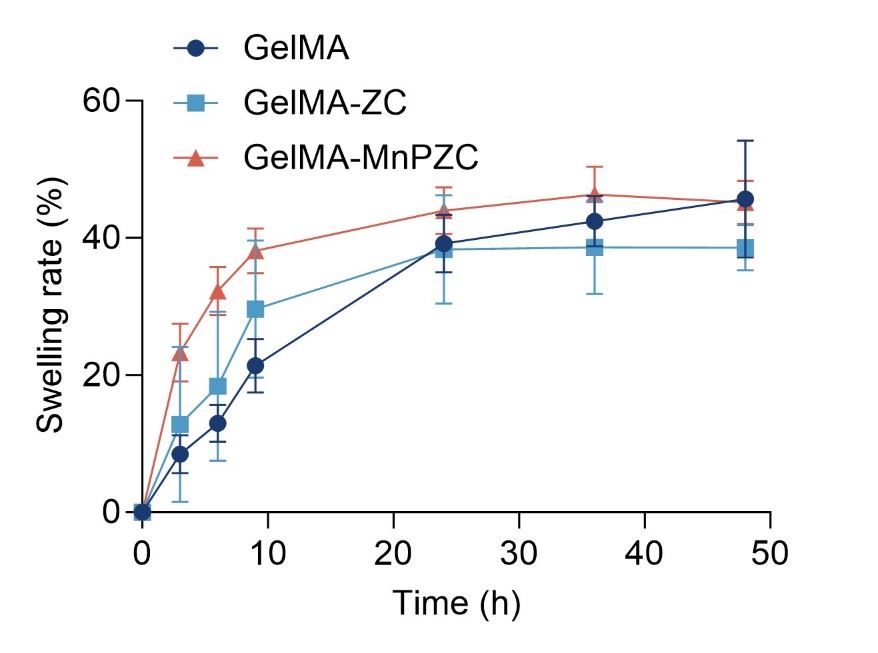


Fig. S6. Swelling behavior of the hydrogel in the simulated diabetic wound exudate.


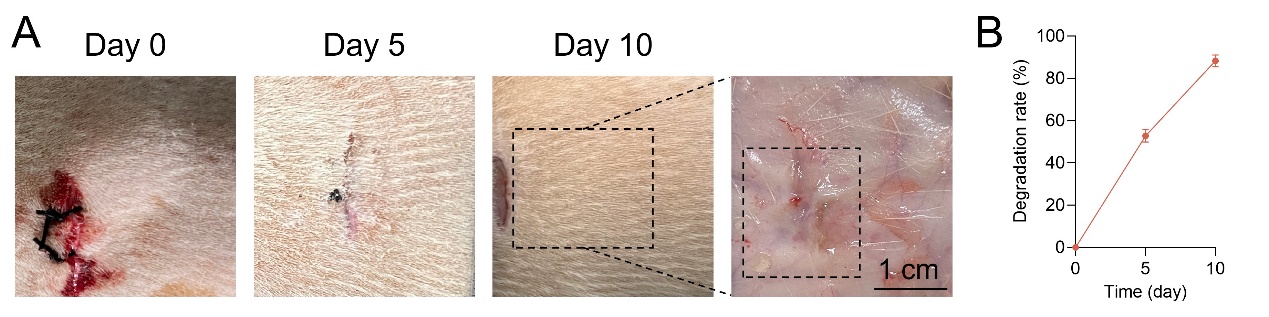


Fig. S7. In vivo degradation images (A) and quantitative analysis of hydrogels (B).


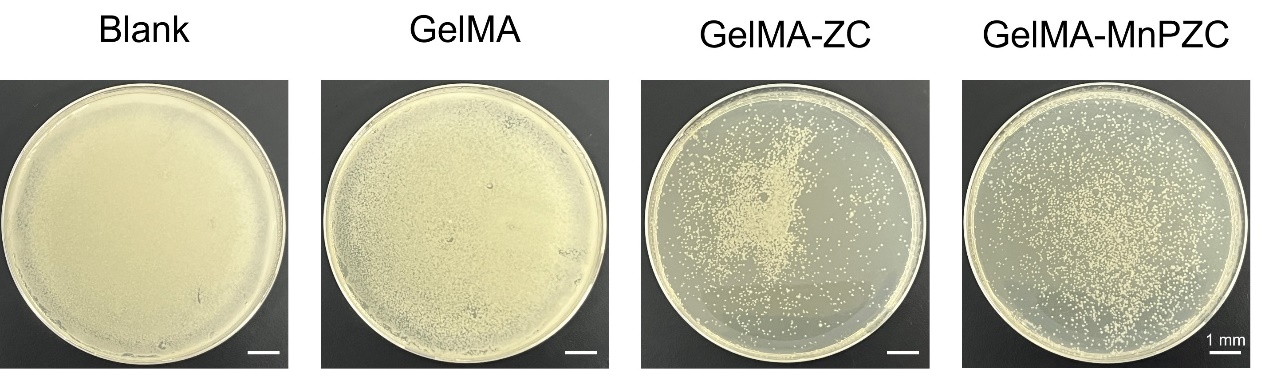


Fig. S8. In vivo antibacterial properties of hydrogels.


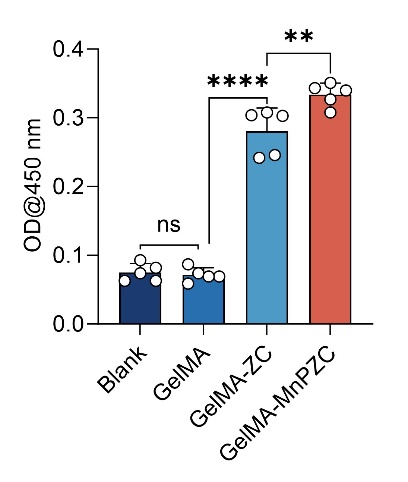


Fig. S9. HaCat cell proliferation on different hydrogels for 3 days.


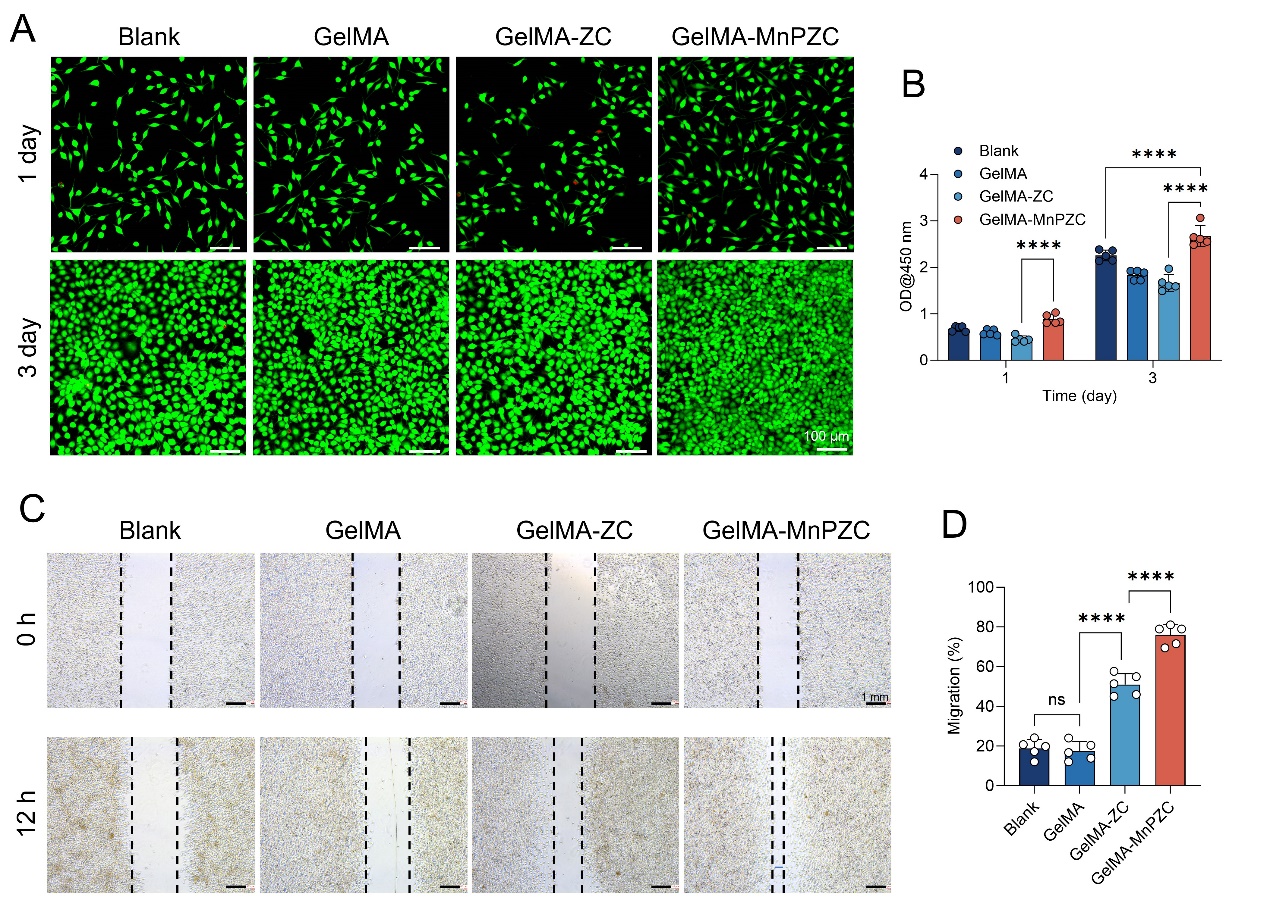


Fig. S10. (A) Representative live/dead staining images and (B) proliferation of L929 cell on different hydrogels in a hypoxic environment for 1 and 3 days. (C) Representative phase-contrast images and (D) quantitative analysis of L929 cells migration to the scratched area on different hydrogels.


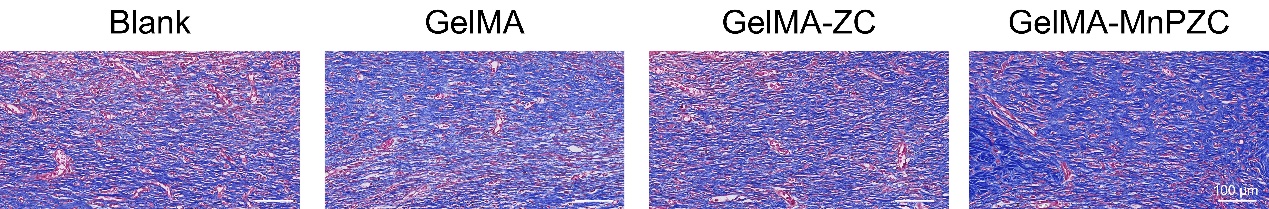


Fig. S11. Representative Masson-stained images of wound sites from different groups on day 15.


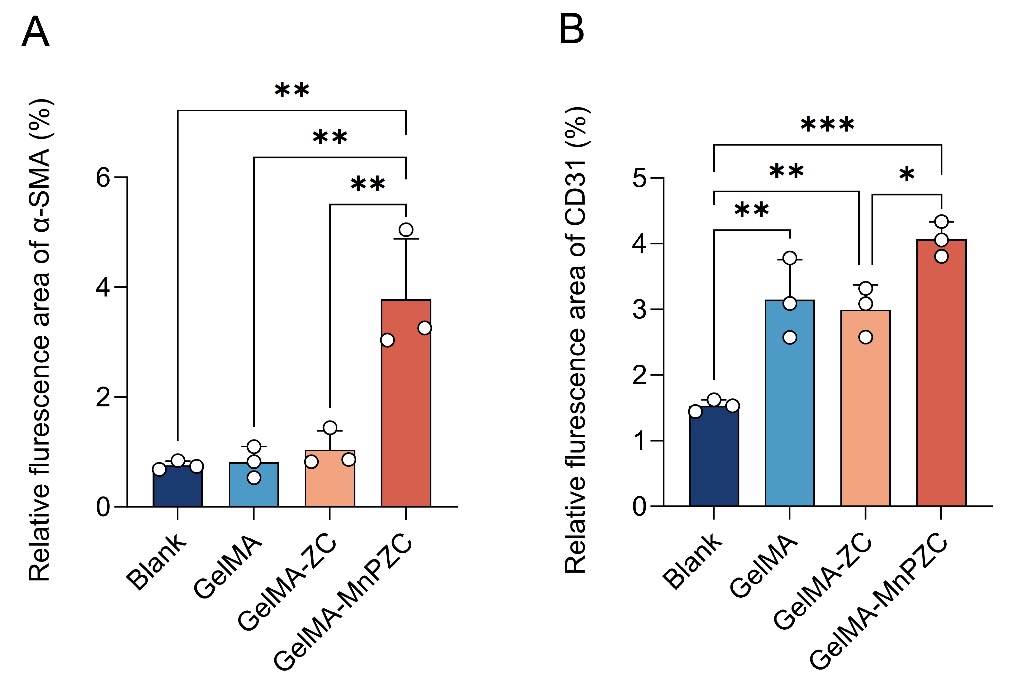


Fig. S12. Relative fluorescence intensity of CD31 (A) and α-SMA (B).


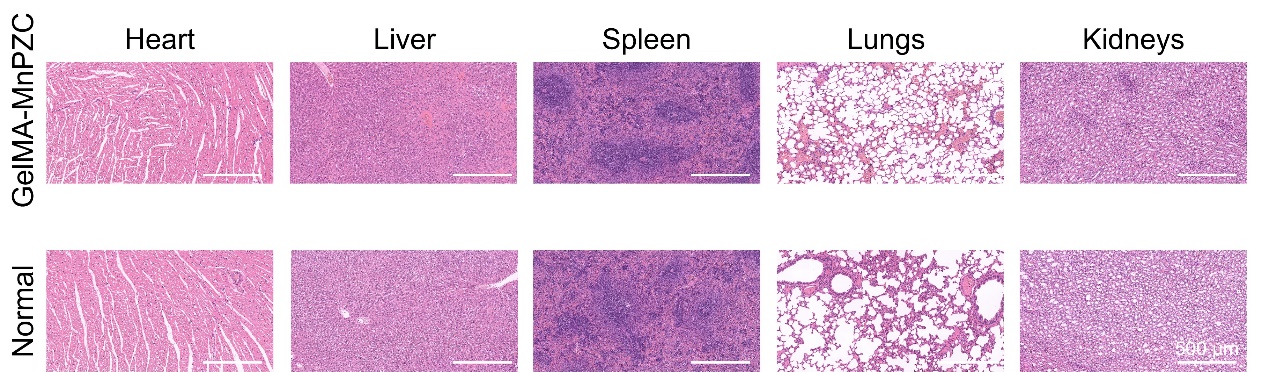


Fig. S13. In vivo biosafety, H&E slice images of major organs.
